# A Temporal Phospho-acetylome Atlas of Human Myogenesis Identifies coordinated Post-Translational Regulation

**DOI:** 10.1016/j.mcpro.2026.101619

**Published:** 2026-07-02

**Authors:** Lauren E. Smith, Christina E. Hagensen, Vasileios Tsiamis, Ashley J. van Waardenberg, Kamel Mamchaoui, Ole N. Jensen, Veit Schwämmle, Adelina Rogowska-Wrzesinska

**Affiliations:** 1 Department of Biochemistry and Molecular Biology, University of Southern Denmark, Odense, Denmark; 2 Australian Institute of Tropical Health and Medicine, James Cook University, Cairns, Australia; 3 Sorbonne University, INSERM, Institute of Myology, Center for Research in Myology, Paris, France

**Keywords:** skeletal muscle differentiation, phosphorylation, lysine acetylation, post-translational modification crosstalk, quantitative proteomics, kinase activity prediction, lamin A/C, GAPDH

## Abstract

Skeletal muscle differentiation depends on precise temporal regulation of protein modifications. To define how phosphorylation and lysine acetylation change during this process, we applied tandem mass tag (TMT)-based quantitative proteomics to human myoblasts sampled at six stages spanning proliferation, induction of differentiation, and early myotube formation. Using sequential enrichment of phosphorylated and acetylated peptides, high-pH fractionation, and high-resolution mass spectrometry, we identified more than 22,000 modified peptides and quantified their temporal behavior after correction for protein abundance. Phosphorylation exhibited extensive site-specific remodeling throughout the time course, whereas acetylation showed a pronounced relative increase during late differentiation. Integration of corrected modification levels with protein abundances and temporal clustering revealed stage-specific regulation of processes linked to cell-cycle withdrawal, metabolic transitions, cytoskeletal reorganization, and chromatin-associated functions. Predicted temporal activity profiles of kinases, acetyltransferases, and deacetylases uncovered coordinated regulatory patterns, including activity relationships involving CSNK2A1-HDAC1/2, PRKAA1-HAT1, and CDK1/2-KAT7. Dual-modified proteins such as lamin A/C and glycolytic enzymes display densely regulated clusters of phosphorylation and acetylation sites that may contribute to nuclear remodeling and metabolic adaptation during myogenesis. Together, this work provides a high-resolution temporal phospho-acetylome atlas of human muscle cell differentiation, identifies candidate phosphorylation-acetylation coordination patterns, and establishes a systems-level resource for future mechanistic studies of post-translational regulation in skeletal muscle development.

## Highlights

• Temporal phospho-acetylome mapped across six stages of human myogenesis.
• Acetylation rises late, contrasting with dynamic early and mid-stage phosphorylation.
• Predicted kinase and acetylation enzyme activities show coordinated regulation.
• Dual-modified proteins, lamin A/C and GAPDH, highlight candidate PTM integration regions.
• Protein-corrected PTM quantification provides a high-resolution temporal atlas.

## Graphical Abstract

**Figure:**
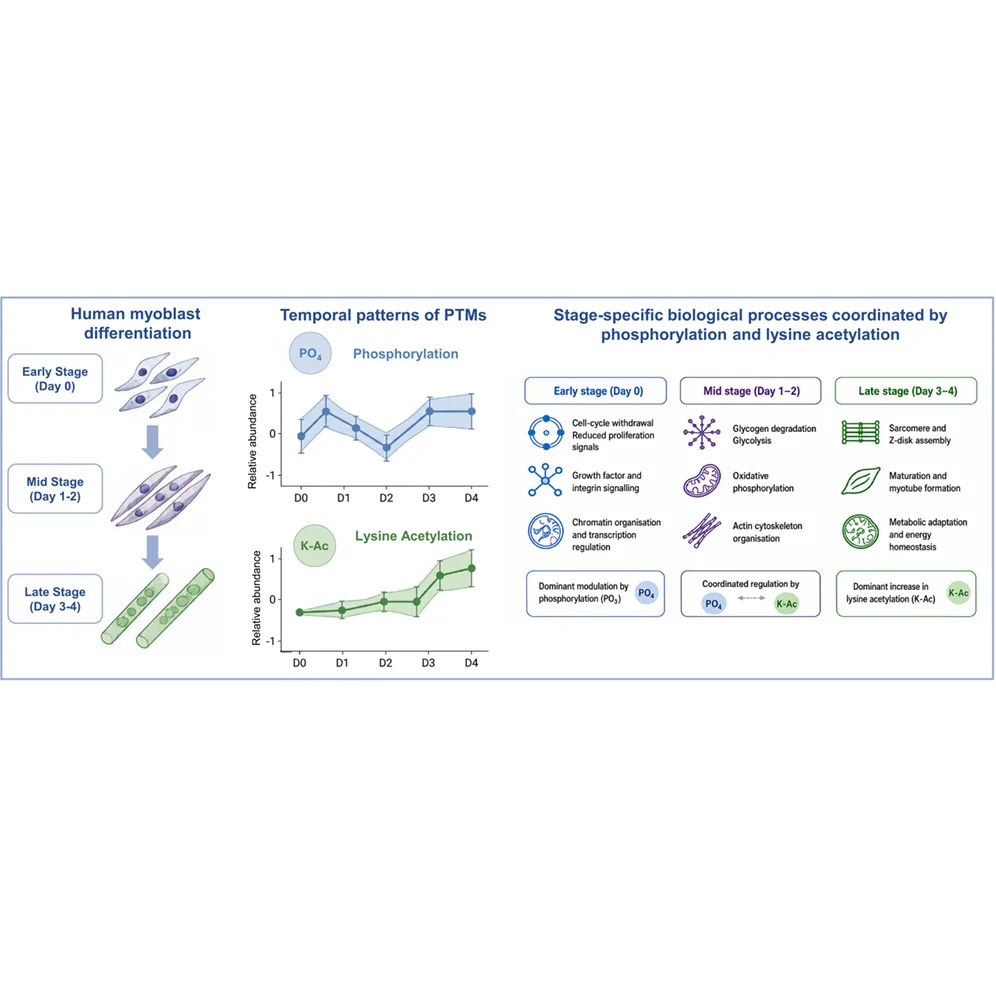

## In Brief

This study presents a comprehensive temporal phospho-acetylome of human myogenesis by integrating TMT-based proteomics, PTM enrichment, protein-level correction, and enzyme activity prediction. Phosphorylation displays dynamic regulation throughout differentiation, whereas acetylation increases sharply at late stages. Coordinated enzyme activity patterns and dual-modified proteins, including lamin A/C and glycolytic enzymes, reveal potential PTM coordination patterns. The dataset provides a detailed framework for understanding post-translational control during muscle development.

Skeletal muscle differentiation is a highly coordinated process that transforms proliferating myoblasts into multinucleated myotubes. This transition requires precise temporal control of transcriptional programs, metabolic reorganization, cytoskeletal assembly, and cell-cycle withdrawal. Central regulators such as MyoD, MEF2, MYF5, FOXO1, and PGC-1α integrate signals from multiple pathways, including MAPK, PI3K-AKT, AMPK, and CaMK networks, to drive lineage commitment and maturation (1). Although the transcriptional and signaling determinants of myogenesis have been studied extensively, the systems-level temporal coordination of post-translational modifications (PTMs) during differentiation remains incompletely resolved.

Recent transcriptomic, proteomic, and phosphoproteomic studies have substantially advanced the understanding of skeletal muscle differentiation and identified large-scale molecular changes accompanying myogenic progression (2, 3). Previous proteomic analyses have characterized broad protein abundance remodeling during differentiation, while more recent phosphoproteomics (4) and glycoproteomic studies (5) have revealed dynamic regulation of signaling pathways, cytoskeletal organization, and membrane-associated processes during myotube formation. However, most studies have focused on individual PTM classes, limited differentiation stages, or specific regulatory pathways, and therefore do not resolve how phosphorylation and lysine acetylation evolve in a coordinated temporal manner across the differentiation trajectory. In particular, the relationship between PTM dynamics, remodeling of protein abundance, and coordinated regulation of kinases and acetylation enzymes during human myogenesis remains insufficiently understood.

Phosphorylation and lysine acetylation are two major PTM classes that regulate protein stability, enzymatic activity, chromatin accessibility, transcription factor function, and metabolic flux. Individual phosphorylation events on key myogenic regulators such as MyoD, MEF2, and FOXO transcription factors have been shown to influence the differentiation stage or activity (6, 7). Acetylation also modulates chromatin structure, metabolic enzymes, and transcriptional regulators relevant to muscle development, and several acetyltransferases and deacetylases, including KAT2A, p300, SIRT1, and SIRT2, have been implicated in myogenesis and regeneration (8, 9, 10, 11). However, most studies have examined single proteins or specific regulatory nodes, leaving unclear how phosphorylation and acetylation collectively evolve across the differentiation timeline.

Moreover, phosphorylation and acetylation do not act independently. Crosstalk between these modifications has been described for transcription factors, metabolic enzymes, and nuclear scaffolding proteins, where combinations of PTMs can act cooperatively or antagonistically to fine-tune protein function (12). Kinases and acetylation-associated enzymes are themselves often regulated by phosphorylation, suggesting that coordinated activity between these enzyme classes may shape differentiation through layered PTM control. Despite these emerging concepts, no study has defined how phosphorylation and acetylation are temporally coordinated during human myogenesis, nor how enzyme activities and potential PTM crosstalk relationships shift across the differentiation trajectory.

A comprehensive temporal map of phosphorylation and lysine acetylation is therefore required to resolve how these modification classes contribute to the ordered sequence of biological events that define myogenesis. Differentiation involves stepwise transitions in transcriptional activity, metabolism, cytoskeletal organization, and nuclear architecture, and each transition depends on precise PTM-mediated control. Existing studies typically focus on individual proteins, single PTM types, or limited time points, and thus cannot capture coordinated regulation across signaling, chromatin, and metabolic networks. A temporal, dual-PTM proteomic approach is essential to identify stage-specific modification events, determine whether kinases and acetylation-related enzymes show aligned activity patterns, and reveal potential points of phosphorylation-acetylation coordination that are not observable in static or single-modality datasets.

During myogenesis, proliferating myoblasts first undergo cell-cycle withdrawal and lineage commitment before entering early differentiation programs characterized by cytoskeletal remodeling, metabolic adaptation, cellular alignment, and eventual fusion into multinucleated myotubes. These transitions involve coordinated changes in signaling activity, chromatin regulation, energy metabolism, and structural organization, all of which depend on tightly regulated post-translational control. Temporal resolution is therefore essential for distinguishing early signaling events associated with proliferation arrest from later processes linked to myotube maturation and contractile apparatus assembly (1).

Here, we address this gap by generating a comprehensive temporal map of phosphorylation and lysine acetylation during human myoblast differentiation. Using deep tandem mass tag (TMT)-based proteomics, combined phosphorylated and acetylated peptide enrichment, and protein abundance correction using VIQoR (13), we quantified more than 22,000 modified peptides across six defined differentiation stages. We integrated these data with statistical modeling, pathway enrichment, variance-sensitive clustering, and enzyme activity predictions using KinSwing (14) to resolve the temporal regulation of kinases, acetyltransferases, and deacetylases. We further examined dual-modified proteins and mapped regulated sites onto structural domains to identify candidates for coordination of phosphorylation and acetylation.

This work provides the most extensive temporal phospho-acetylome of human myogenesis to date. We reveal distinct stage-specific dynamics of phosphorylation and acetylation, predict coordinated enzyme activity programs across the differentiation time course, and identify candidate PTM crosstalk nodes, including glycolytic enzymes and lamin A/C, that may integrate metabolic and nuclear remodeling processes. Together, these findings offer a systems-level framework for understanding post-translational regulation during skeletal muscle development and generate mechanistic hypotheses relevant to muscle biology, regeneration, and disease.

## Experimental Procedures

### Cell Culture and Differentiation of Human Myoblasts

Human immortalized satellite cells (KM155C25) were obtained from the Institute of Myology (Sorbonne Université) and cultured following established protocols (15, 16). Cells were maintained in growth medium containing DMEM supplemented with Medium 199, fetal bovine serum, fetuin, hEGF, bFGF, insulin, and penicillin/streptomycin. Differentiation was induced at confluence by replacing the growth medium with differentiation medium consisting of DMEM with insulin and penicillin/streptomycin.

Cells were harvested at six stages: proliferating cultures at 70% confluence (D − 1), confluent myoblasts (D0), and cells at 24 h, 48 h, 72 h, and 96 h after induction of differentiation (D1-D4). All samples were collected in three independent biological replicates, each grown and processed separately. Detailed medium compositions, reagent sources, and stepwise procedures are provided in the Supplementary Methods.

### Protein Extraction, Digestion, and TMT Labeling

Cells were lysed in TEAB-based buffer (pH 8.5) containing SDC, TCEP, chloroacetamide, and protease/phosphatase inhibitors. Lysates were sonicated, heated, and clarified, and protein concentration was determined by tryptophan fluorescence (17).

Proteins (350 μg per sample) were digested using the FASP method (18) employing in-house methylated trypsin (19). A primary overnight digestion (1:25 enzyme: protein) was followed by a secondary 3-h digestion (1:50). Peptides were desalted on HLB cartridges and lyophilized.

Peptides (100 μg) were labeled using TMT10plex reagents (Thermo Fisher Scientific) according to the manufacturer’s instructions. Two TMT10plex sets were assembled, each including a pooled mixed reference channel (131) for cross-plex normalization. The multiplex layout and sample assignment are described in the Supplementary Methods.

### Enrichment of Phosphorylated and Acetylated Peptides

A sequential enrichment workflow was used to isolate phosphorylated and lysine-acetylated peptides. TMT-labeled peptides were first subjected to TiO_2_ enrichment using multiple bead incubations in glycolic acid-based loading buffer. Phosphorylated peptides were eluted with ammonium hydroxide, dried, and purified on C18/POROS R3 micro-columns.

The combined TiO_2_ supernatants were subsequently enriched for lysine-acetylated peptides using the PTMScan Acetyl-Lysine Motif Kit (Cell Signaling Technology #13416). Immunoaffinity-purified peptides were eluted under acidic conditions and desalted on C18/POROS R3 columns.

Complete reagent compositions, bead quantities, wash steps, and incubation conditions are provided in the Supplementary Methods.

### High-pH Fractionation and LC-MS/MS Analysis

Phosphorylated and non-modified peptide fractions were separated using high-pH reverse-phase chromatography on a Dionex UltiMate 3000 system. 20 concatenated fractions were collected, dried, and reconstituted for LC-MS/MS analysis.

Peptides were analyzed on a Q Exactive HF-X Orbitrap mass spectrometer (Thermo Fisher Scientific) coupled to a nanoLC system equipped with a 50-cm × 75-μm C18 analytical column operated at 50°C. Acetylated and non-modified peptides (not enriched for PTMs and used only for protein-level quantification) were separated using a 55-min gradient (8–45% ACN, 0.01% FA), and phosphorylated peptides using a 5 to 45% gradient.

MS1 spectra were acquired at 120,000 resolution (AGC target 3 × 10^6^, max IT 50 ms). MS2 spectra were acquired using a Top20 DDA method at 45,000 resolutions (AGC target 1 × 10^5^, max IT 100 ms) with HCD at NCE 32 (acetylated/non-modified) or 34 (phosphorylated). Additional chromatographic and mass spectrometry parameters are provided in the Supplementary Methods.

### Proteomics Data Processing

Raw files were processed using Proteome Discoverer 2.3 (Thermo) with Mascot as the search engine against the SwissProt human database (taxonomy 9606; release March 2020; 20,431 protein entries). Search parameters included precursor mass tolerance of 10 ppm, fragment tolerance of 0.05 Da, trypsin specificity with up to four missed cleavages, fixed carbamidomethylation of cysteine, and variable modifications including phosphorylation (S, T, Y), lysine acetylation, and TMT10plex labeling on peptide N-termini and lysines.

Peptides were filtered using Percolator to achieve PSM FDR <1%. Modification site localization was assigned using ptmRS, retaining sites with localization probability ≥90%. Reporter-ion intensities were quantified using a 30-ppm integration window. PSMs lacking reference channel intensity or containing shared quantification labels were removed. Reporter ion quantification was performed using the Proteome Discoverer Reporter Ions Quantifier node with HCD MS/MS-based TMT quantification. Reporter abundance calculation used the Proteome Discoverer automatic reporter abundance mode. An average reporter signal-to-noise threshold of 6 was applied, and the co-isolation threshold was set to 100. Isotopic impurity correction was not applied during reporter ion quantification. Protein-level quantification was based on unique peptides only using protein-group-based peptide assignment.

Data from the two TMT plexes were initially normalized by subtracting log_2_-transformed intensities of the pooled reference channel and subsequently combined. The resulting log-transformed quantitative data were further processed in VIQoR using median normalization, with 18 quantitative columns across six conditions and strict protein inference settings. Additional filtering and quality control steps are described in the Supplementary Methods.

### Protein Inference and Protein-Normalized PTM Correction

Protein inference and correction of modified peptide abundances for protein expression changes were performed using VIQoR (13). VIQoR applies probabilistic peptide-protein assignment, weighted averaging, replicate-aware modeling of missing data, and correction of PTM abundances for underlying protein-level variation. Parameter settings and workflow details are provided in the Supplementary Methods. Protein-abundance correction transforms modified peptide measurements from absolute peptide abundance values toward relative modification levels normalized to the corresponding protein abundance. Therefore, corrected PTM values should be interpreted as protein-normalized modification changes, not as absolute modified peptide copy-number changes. Apparent inverse relationships between protein abundance and corrected PTM values may therefore reflect altered apparent relative occupancy or protein-normalized PTM abundance, although absolute site occupancy was not directly measured.

### Experimental Design and Statistical Rationale

Three biological replicates were analyzed at each of the six time points, resulting in 18 samples across two TMT10plex experiments sharing a common reference channel.

Differential regulation was assessed using the Polystest statistical framework (20). Missing values were handled using the Miss test to account for non-random missingness. Mean coefficient of variation across replicates was ≤12% for all peptide classes; therefore, a ±1.3-fold change threshold was applied. Statistical significance was defined as peptide- or protein-level FDR <0.002. This threshold was selected to maintain an estimated dataset-level false discovery rate of approximately 1% after multiple testing correction.

Analyses were performed in R, where applicable, with examples provided in the Supplementary Methods.

### Clustering, Gene Ontology, and Pathway Analyses

Temporal clustering of proteins and modified peptides was performed using variance-sensitive fuzzy c-means clustering (VSClust; (21, 22)). Enriched Gene Ontology terms were identified using DAVID (23) with Benjamini-Hochberg adjusted *p* ≤ 0.05.

Ingenuity Pathway Analysis (IPA, Qiagen) was used to identify enriched signaling and metabolic pathways using right-tailed Fisher’s exact tests with Benjamini-Hochberg correction (*p* ≤ 0.01). Pathway lists and enrichment scores are provided in Supplementary Table S4.

### Enzyme Activity Prediction Using KinSwing

Kinase, acetyltransferase, and deacetylase activity profiles were predicted using the KinSwingR algorithm (14, 24). Kinase–substrate relationships were derived from PhosphoSitePlus (25), and acetyltransferase/deacetylase substrate relationships were obtained from a curated enzyme-specific lysine acetylation dataset (26). KinSwing is a network-based approach that infers relative enzyme activity by integrating temporal changes at known substrate modification sites. For each enzyme, position weight matrices are constructed from curated substrate motifs and combined with observed modification changes to calculate weighted regulatory scores across the motif–substrate network. Final scores are z-transformed to enable comparison between time points, and statistical significance is determined by randomly perturbing the motif–substrate network 1000 times. KinSwing scores reflect relative changes in predicted enzyme activity rather than absolute biochemical activity. These predictions are based on known substrate annotations and motif-based inference and therefore represent probabilistic estimates of regulatory trends. Scores were calculated for each differentiation time point relative to Day −1, and enzymes with significant predicted activity changes (*p* ≤ 0.05) were retained for downstream analysis. Enzymes exhibiting similar temporal activity trajectories were grouped by hierarchical clustering. Complete KinSwing scores and clustering results are reported in Supplementary Table S8.

## Results

### Global Dynamics of Phosphorylation and Acetylation During Differentiation

**Fig. 1:**
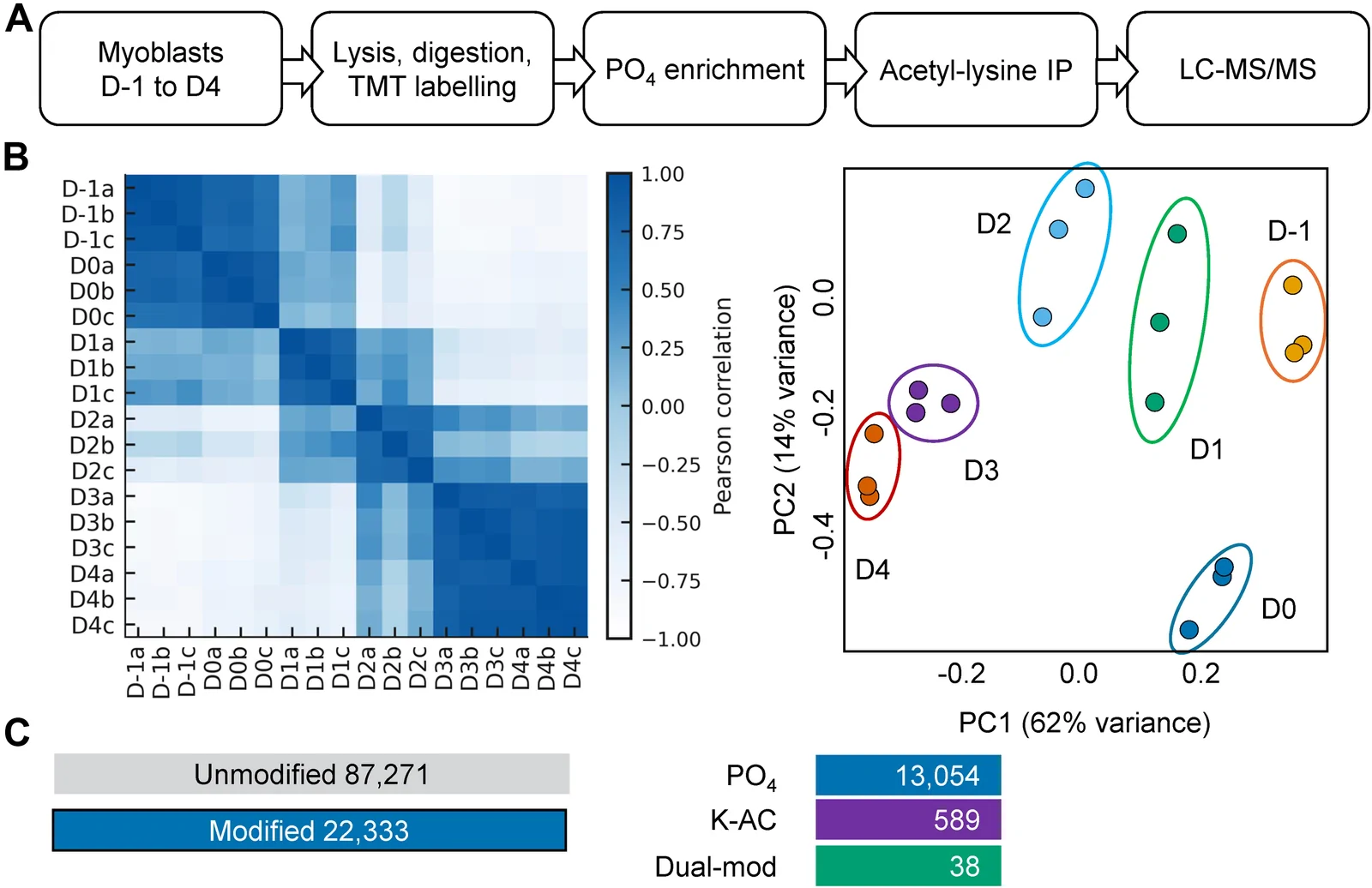
**Workflow, sample overview, and global identification statistics.***A*, overview of the experimental workflow for temporal profiling of phosphorylation and lysine acetylation. Human myoblasts were collected at six stages (D − 1 to D4), followed by lysis, digestion, TMT labelling, enrichment of phosphorylated peptides and lysine-acetylated peptides, and LC-MS/MS analysis. *B*, Pearson correlation heat map of all quantified peptides across biological replicates (*left*) and principal component analysis (*right*) showing separation of samples by differentiation stage. *C*, summary of identified peptides, including the number of unmodified peptides, total modified peptides, and counts of phosphorylated (PO_4_), lysine-acetylated (K-Ac), and dual-modified peptides that were corrected for underlying protein abundance.

To define how post-translational modifications change during myogenesis, cells were collected at six stages spanning D-1 to D4 and processed using the sequential enrichment workflow illustrated in Figure 1*A*. This approach enabled quantitative analysis of phosphorylation and lysine acetylation across the differentiation timeline. Principal component analysis confirmed clear separation of all time points and demonstrated excellent reproducibility among biological replicates (Fig. 1*B*), with low coefficients of variation across peptide classes (Supplementary Table S1).

Across all time points, we identified 109,604 peptides, of which 22,333 carried post-translational modifications with localization probabilities of at least 90% (Fig. 1*C*). These included 21,316 phosphorylated peptides, 949 acetylated peptides, and 68 dual-modified peptides bearing both phosphorylation and lysine acetylation. Dual-modified peptides refer to individual peptide species carrying both phosphorylation and lysine acetylation simultaneously, whereas dual-modified proteins denote proteins identified with both modification classes across different peptide regions. Modified peptide intensities were corrected for underlying protein abundance using VIQoR, and peptides passing localization and correction criteria were retained for downstream analysis (Supplementary Table S2*A*), while corresponding non-corrected modified peptide abundance values are provided in Supplementary Table S2*B*. Consequently, the corrected PTM values reflect protein-normalized relative modification levels. For proteins undergoing strong abundance remodeling during differentiation, corrected PTM profiles can therefore differ from, or even oppose, the corresponding protein abundance profiles. This resulted in 13,681 modified peptides (Supplementary Fig. S1), of which 6667 showed significant regulation (|log_2_ fold change| ≥ 0.38 and FDR <0.002). In parallel, at the protein level, we quantified 6863 protein groups, of which 5868 showed significant abundance changes at least once during differentiation.

**Fig. 2:**
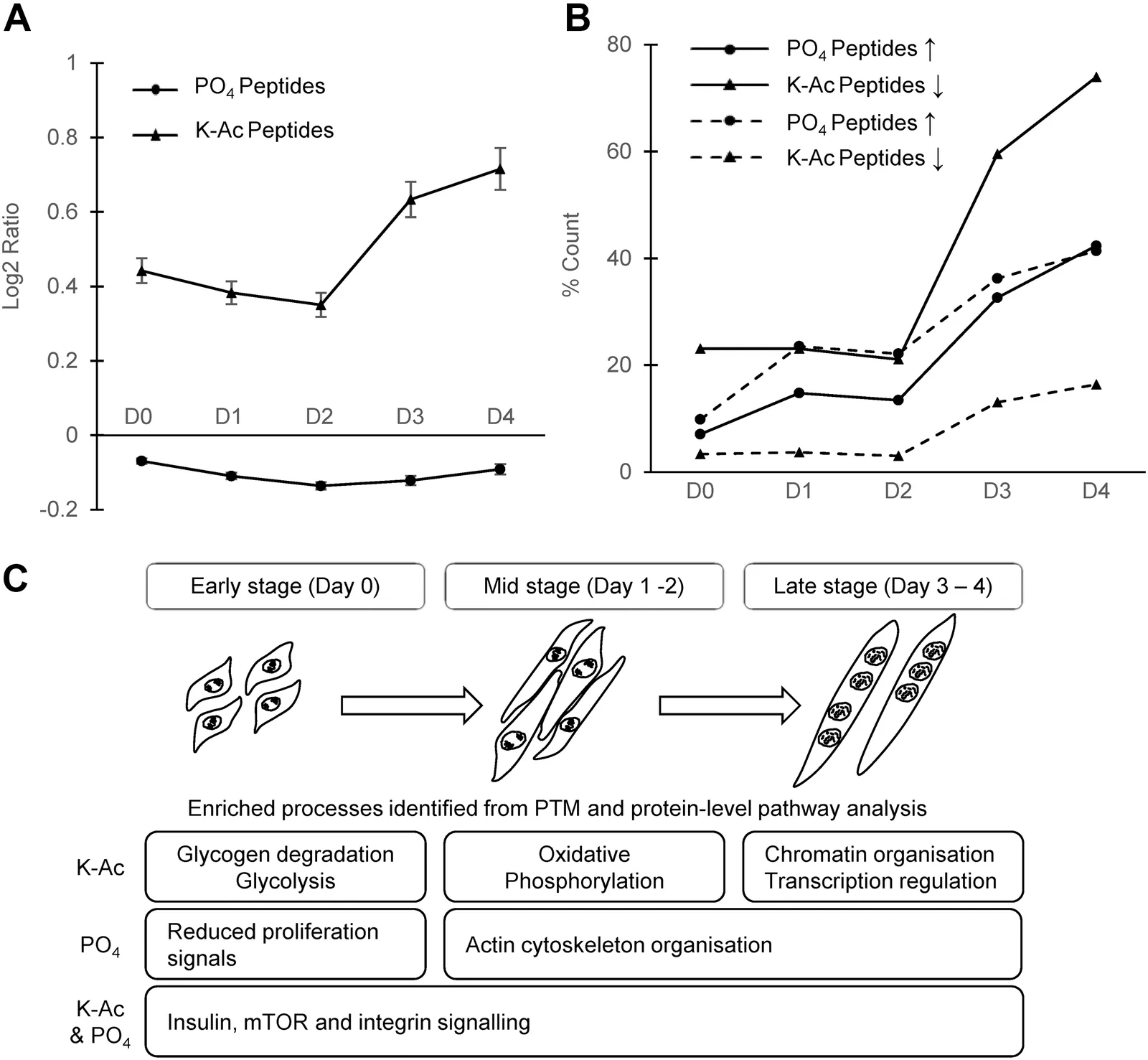
**Temporal regulation of phosphorylation and lysine acetylation during myogenesis.***A*, average log_2_-transformed abundance of significantly regulated phosphorylated peptides (PO_4_) and lysine-acetylated peptides (K-Ac) at each differentiation stage relative to Day −1. Error bars represent the standard error of the mean across biological replicates. *B*, percentage of significantly increased or decreased phosphorylated and lysine-acetylated peptides at each time point, normalized to the total number of quantified modified peptides within each category. Solid and dashed lines indicate increased and decreased peptides, respectively. *C*, schematic overview of early (Day 0), mid (Days 1–2), and late (Days 3–4) differentiation stages, with enriched biological processes identified from pathway analysis of regulated proteins and post-translationally modified peptides. Processes are grouped according to predominant association with lysine acetylation (K-Ac), phosphorylation (PO_4_), or both modification types.

Volcano plot analysis demonstrated progressive increases in regulated proteins, phosphorylated peptides, and lysine-acetylated peptides across the differentiation time course (Supplementary Fig. S2, *A*–*C*), reflecting the extensive molecular remodeling underlying myotube formation. Phosphorylated peptides showed widespread bidirectional changes, whereas acetylated peptides displayed a marked increase in abundance at late time points (Fig. 2, *A* and *B*). These observations indicate distinct temporal patterns: acetylation surges at D3-D4, while phosphorylation shows dynamic regulation throughout the time course. This late-stage increase in lysine acetylation prompted further investigation of acetylation-associated enzyme activity and potential coordination with phosphorylation.

### Pathway- and Cluster-Level Insights into Stage-Specific Regulation

To relate these modification dynamics to biological processes, significantly regulated proteins and modified peptides were analyzed using pathway enrichment and temporal clustering. Ingenuity Pathway Analysis highlighted enrichment of pathways related to cell-cycle exit, metabolic transitions, and cytoskeletal organization (Fig. 2*C*; Supplementary Figs. S3–S7). Variance-sensitive clustering of proteins, phosphorylated peptides, and acetylated peptides revealed groups of co-regulated species associated with distinct stages of myogenesis. Early stages were characterized by regulation of cell-cycle pathways, mid-differentiation by metabolic processes, and late stages by remodeling of cytoskeletal and contractile proteins. These stage-specific patterns are consistent with the known progression of myogenesis from proliferating myoblasts through cell-cycle withdrawal and metabolic reprogramming toward structural maturation and myotube formation. The early decline in cell-cycle-associated signaling and the later increase in cytoskeletal and contractile organization pathways therefore recapitulate established biological transitions during skeletal muscle differentiation. The recovery of these known regulatory programs supports the biological validity of the temporal PTM, and protein abundance patterns observed in the dataset. Together, these analyses link temporal PTM patterns to the major functional transitions that define myoblast differentiation.

### Structural Components of Myogenesis Reflect Coordinated Protein and PTM Changes

**Fig. 3:**
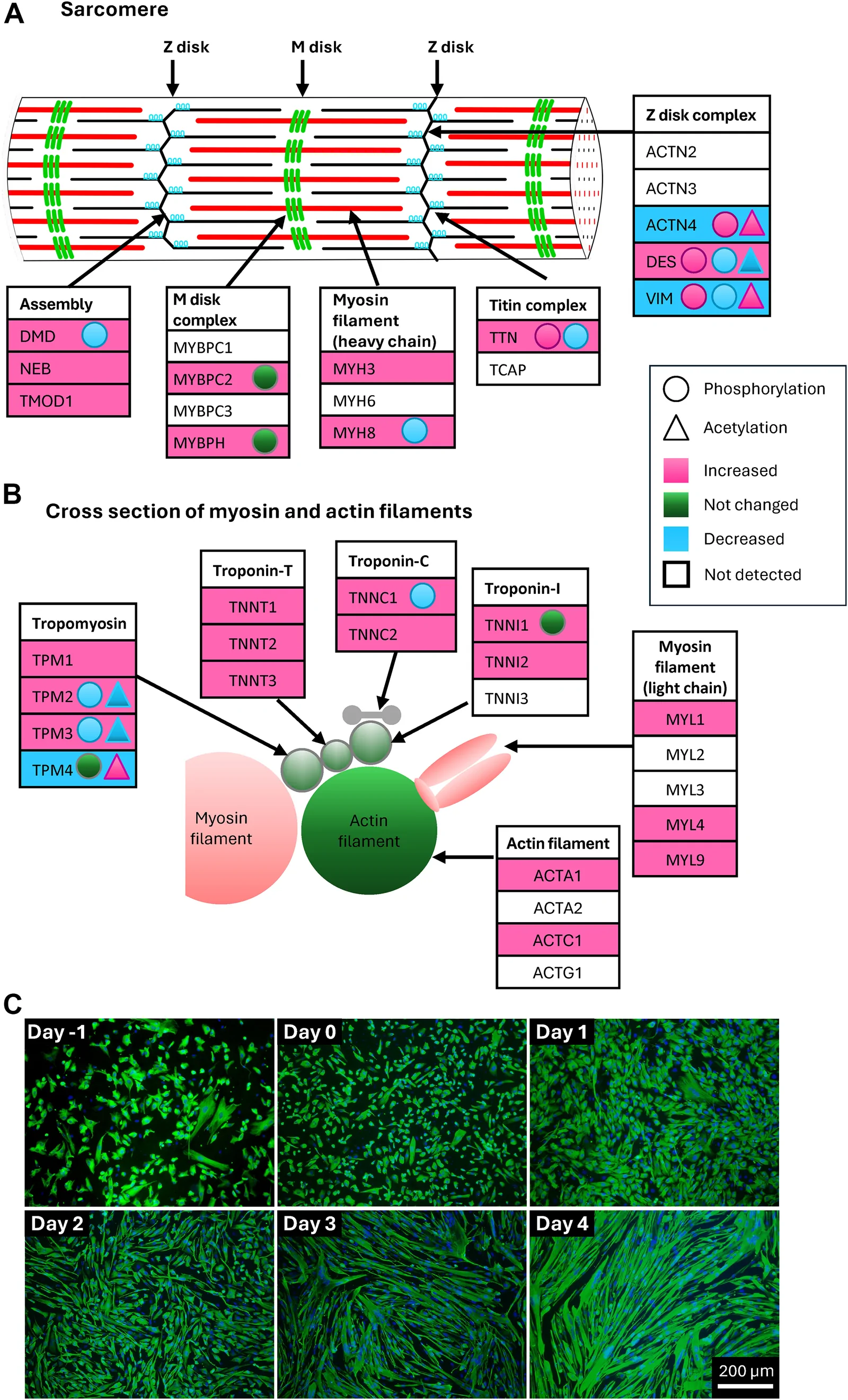
**Protein abundance and post-translational modifications are mapped to structural components of developing muscle.***A-B*, protein abundance changes and identified phosphorylation and lysine-acetylation sites were mapped to major components of the striated muscle contractile machinery. Proteins are colored by abundance change (*pink*, increased; *blue*, decreased; *grey*, unchanged). Phosphorylation sites are indicated by circles and acetylation sites by triangles. *C*, immunostaining of desmin (*green*) and nuclei (DAPI, *blue*) from Day −1 to Day 4 showing morphological progression during differentiation. Scale bar: 200 μm.

Changes in protein abundance and PTMs were mapped onto structural components of developing muscle fibers (Fig. 3, *A* and *B*). Proteins associated with the Z-disk and sarcomeric structures increased steadily during differentiation and exhibited extensive phosphorylation and acetylation. Immunofluorescence staining of desmin corroborated the increase in structural protein abundance and demonstrated the expected morphological transition from mononucleated myoblasts to multinucleated myotubes (Fig. 3*C*). These observations connect PTM and protein-level changes to the architectural reorganization of myofibrils during early myogenesis.

The progressive accumulation of sarcomeric and Z-disk-associated proteins is consistent with the transition from proliferating myoblasts to aligned, structurally mature myotubes. The coordinated regulation of phosphorylation and acetylation across these structural components further suggests that PTM remodeling contributes to the ordered assembly and stabilization of contractile architecture during differentiation.

### Predicted Enzyme Activities Underlying PTM Regulation

**Fig. 4:**
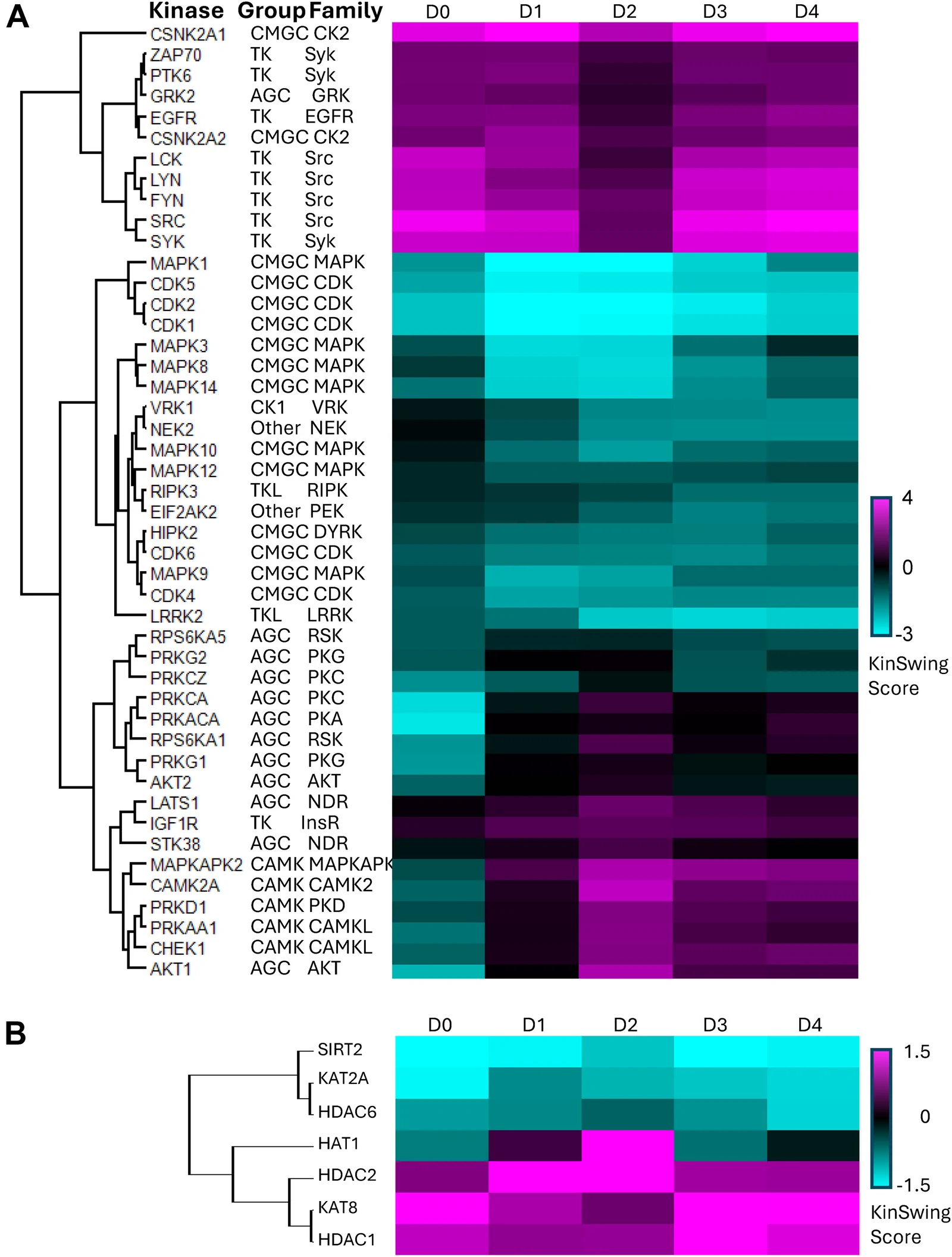
**Predicted kinase and acetylation-related enzyme activities across the differentiation timeline.***A*, heat map of KinSwing scores for kinases across differentiation stages. Scores represent predicted activity changes relative to D-1 and were filtered for *p* ≤ 0.05. Colors indicate increased (*pink*) or decreased (*green*) predicted activity. Kinase groups and families are indicated. *B*, heat map of predicted activity changes for acetyltransferases and deacetylases calculated using the same criteria. Enzymes in both panels are grouped by hierarchical clustering based on similarity of temporal activity profiles.

To investigate whether groups of kinases, acetyltransferases, and deacetylases exhibit coordinated temporal regulation during myogenesis, we predicted enzyme activity profiles using KinSwing scores, which reflect the direction and magnitude of predicted activity changes based on regulated substrate sites and do not represent absolute enzymatic activity. Hierarchical clustering of KinSwing scores revealed several major temporal patterns among kinases, including groups with sustained increased activity, early decreases followed by stabilization, and delayed activation at late differentiation stages, encompassing members of the CMGC (cyclin-dependent kinase and MAP kinase), AGC, and tyrosine kinase families (Fig. 4*A*) (Supplementary Table S8). Only enzymes with significant KinSwing scores (*p* ≤ 0.05) relative to D − 1 were included in the clustering. Kinases with sustained increases included members of the tyrosine kinase and AGC families, such as SRC and AKT isoforms, whereas kinases showing decreasing predicted activity belonged predominantly to the CMGC family, including CDK1 and CDK2.

For acetylation-associated enzymes, most acetyltransferases and deacetylases were separated into two primary clusters representing progressive increases or decreases in predicted activity across the time course (Fig. 4*B*). HAT1 displayed a distinct transient increase at D2, while HDAC1 and HDAC2 showed gradual increases from D − 1 to D4. These predicted activity trajectories were consistent with the temporal patterns observed for phosphorylation and lysine acetylation at the peptide level, supporting the presence of coordinated regulatory changes during differentiation.

The predicted decrease in CDK-family activity, together with increased regulation of acetylation-associated enzymes, is consistent with the transition from proliferative myoblast states toward differentiation-associated chromatin and metabolic remodeling. Likewise, the identification of temporally regulated SRC, AKT, PRKAA1, and HDAC-associated signaling patterns aligns with established pathways implicated in myogenic progression and muscle maturation. The recovery of these known signaling relationships therefore provides additional support for the view that the temporal PTM dynamics captured in this dataset reflect biologically relevant differentiation programs rather than isolated modification events.

### Candidate Phosphorylation-Acetylation Coordination Relationships

We next examined potential points of phosphorylation–acetylation coordination by identifying acetyltransferases and deacetylases with significant predicted activity changes and assessing whether kinases with aligned temporal activity profiles were known to regulate these enzymes (Table 1).

**Table 1:** Candidate phosphorylation–acetylation coordination relationships during myogenesis

Acetylation-associated enzyme	Enzyme activity	PO_4_ site regulation	Candidate kinase	Kinase activity	Concordance	Evidence level	Reference
HDAC2^a^	↑	-	CSNK2A1^a^	↑	Y	Direct	(44, 45)
HDAC1^b^	↑	-	CSNK2A1^b^	↑	Y	Direct	(46)
KAT2A	↓	-	CDK4^b^	↓	Y	Predicted	(47)
SIRT2^b^	↓	-	PRKAA1^b^	↑	Y	Indirect	(48)
SIRT2^b^	↓	-	SRC^b^	↑	Y	Direct	(49)
HAT1	↑	-	PRKAA1^b^	↑	Y	Direct	(50)
HDAC7^bc^	-	S358 ↑	PRKD1^b^	↑	Y	Direct	(51)
KAT7^c^	-	T88 ↓	CDK1^b^	↓	Y	Direct	(52)
			CDK2^b^	↓	Y	Direct	(34)
SIRT1^bc^	NS	S47 ↓	MAPK8^b^	↓	Y	Direct	(53)
		S27 ↑	HIPK2^b^	↓	N	Direct	(54)
		S27 ↑	MAPK8^b^	↓	N	Direct	(55)

Pairs consist of acetyltransferases or deacetylases and candidate kinases identified based on temporally aligned KinSwing-predicted activity profiles. Enzyme activity and kinase activity reflect predicted temporal activity changes across differentiation. Phosphorylation site regulation denotes significantly regulated phosphosites identified in the present proteomics dataset. Symbols: ↑ increased; ↓ decreased; – no detectable change; NS not significant. Concordance indicates agreement between predicted kinase and enzyme activity trends (Y, temporally aligned; N, temporally divergent). Superscripts: a previously reported in muscle-related contexts; b protein implicated in myogenesis; c manually curated pair. Evidence level: Direct, site-mapped phosphorylation demonstrated biochemically; Indirect, functional association without demonstrated phosphorylation; Predicted, inferred from temporally aligned activity patterns in this study.

Several identified pairs correspond to kinase–enzyme relationships previously demonstrated biochemically. These include CSNK2A1–HDAC1/2, PRKAA1–HAT1, PRKD1–HDAC7, CDK1/2–KAT7, and SRC–SIRT2, all of which have reported site-specific phosphorylation events. The concordant temporal activity patterns observed here, therefore, recapitulate established regulatory interactions and support the validity of the KinSwing-based inference approach in the context of myogenesis.

For SIRT2, CDK1, and CDK2 are known to phosphorylate and inhibit enzymatic activity. However, the parallel decrease in both CDK1/2 and SIRT2 predicted activity suggests that CDK-mediated phosphorylation is unlikely to account for SIRT2 regulation during differentiation, indicating that alternative regulatory inputs, including SRC, may predominate.

In addition to these validated interactions, several pairs represent indirect functional associations or novel candidate links. For example, PRKAA1 and SIRT2 displayed opposing temporal activity patterns, consistent with reported pathway-level interactions but without evidence of direct phosphorylation. KAT2A and CDK4 exhibited concordant decreases in predicted activity, suggesting a potential regulatory relationship that has not previously been demonstrated in muscle differentiation.

Collectively, these relationships remain correlative; however, the coexistence of literature-supported kinase–enzyme pairs and novel temporally aligned candidates highlights potential regulatory nodes where phosphorylation and acetylation may act in a coordinated manner during differentiation.

### Case Studies of Dual-Modified Proteins: GAPDH and Lamin A/C

**Fig. 5:**
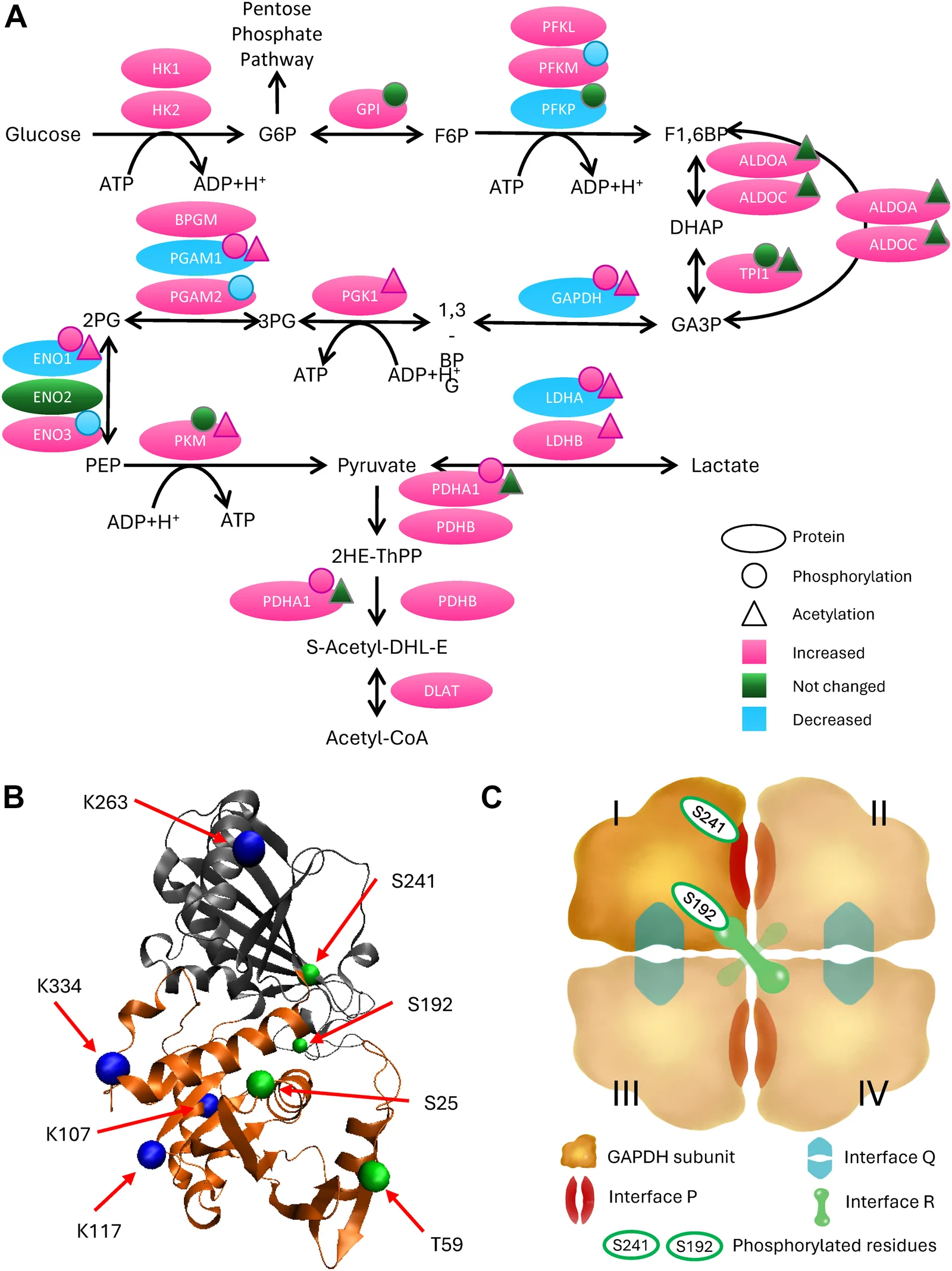
**Post-translational regulation of glycolytic enzymes with focus on GAPDH.***A*, regulated phosphorylation and acetylation sites are mapped to enzymes of the glycolytic pathway. Each enzyme is annotated with increased, decreased, unchanged, or undetected PTM levels at Day 4 relative to Day −1. *B*, three-dimensional structure of the GAPDH subunit (PDB: 1U8F) annotated with identified modification sites. Acetylated residues are shown in dark blue and phosphorylated residues in green. Domains are indicated as NAD^+^-binding (*orange*) and catalytic (*grey*). *C*, simplified representation of the GAPDH tetramer illustrating the three dimer-dimer interfaces: the P interface (*red*), R interface (*green*), and Q interface (*light blue*). Positions of S241 and S192 are shown near the P and R interfaces, respectively.

Among the dual-modified proteins, glycolytic enzymes formed a prominent group, reflecting substantial PTM regulation of central metabolism (Fig. 5*A*; Supplementary Table S9). GAPDH exhibited nine significantly regulated modification sites located across the NAD^+^-binding and catalytic domains (Fig. 5, *B* and *C*). Several sites were positioned near oligomerization interfaces, suggesting potential structural sensitivity to PTM changes during metabolic remodeling.

**Fig. 6:**
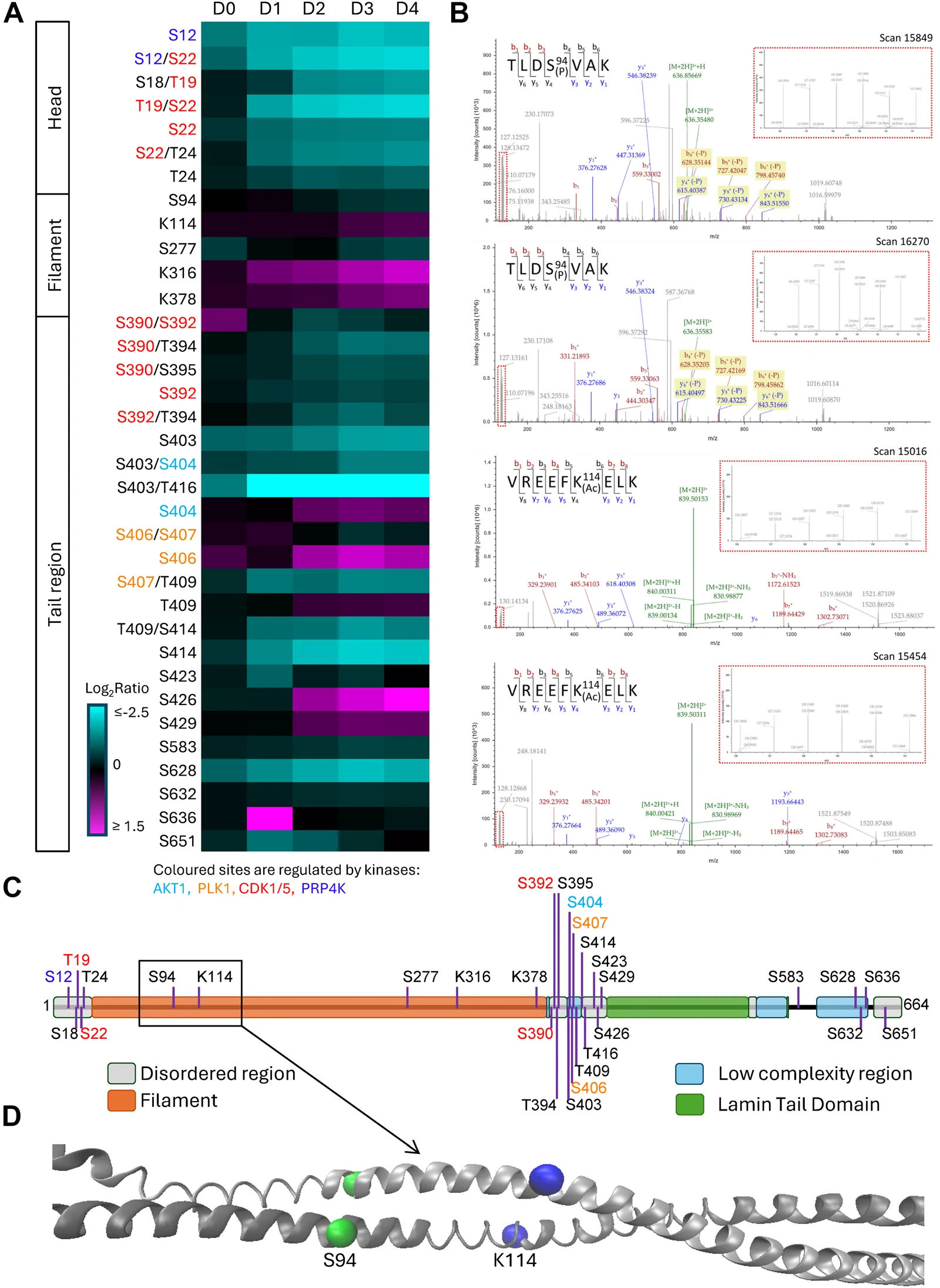
**Phosphorylation and acetylation map of lamin A/C during myogenesis.***A*, heat map of temporally regulated phosphorylation and acetylation sites on lamin A/C across differentiation stages. Colored labels indicate known regulatory kinases annotated in PhosphoSite. *B*, Representative MS/MS spectra illustrating identification of acetylated K144 and phosphorylated S94. *C*, linear domain organization of lamin A/C with all mapped modification sites positioned along head, filament, and tail regions. *D*, structural segment of coiled lamin A/C filaments (PDB: 6JLB) showing spatial proximity of S94 (*green*) and K114 (*dark blue*).

Lamin A/C was the most extensively modified protein identified, with 35 regulated phosphorylation and acetylation sites distributed across the head, filament, and tail regions (Fig. 6, *A*–*C*). Many regulated sites localized to established PTM hotspot regions with extensive prior annotation in PhosphoSitePlus (27), including densely modified phosphorylation clusters within the lamin tail domain. Several phosphorylation sites corresponded to kinases with temporally aligned predicted activity changes and closely positioned phosphorylation-acetylation pairs displayed opposing temporal regulation. Structural mapping further revealed spatial proximity between selected phosphorylation and acetylation sites (Fig. 6*D*). Together, these observations support lamin A/C as a prominent candidate for PTM integration associated with nuclear remodeling during myogenesis.

### Summary of Temporal PTM Regulation

Together, these data reveal extensive and stage-specific regulation of phosphorylation, acetylation, and protein abundance during myoblast differentiation. Global modification trends, pathway-level behavior, predicted regulatory enzyme activities, and analyses of dual-modified proteins converge to show that multiple layers of post-translational regulation act in a coordinated manner during the transition from proliferating myoblasts to early myotubes. These observations motivate the integrative interpretation presented in the Discussion.

## Discussion

### Stage-Specific Coordination Between Phosphorylation and Acetylation During Myogenesis

Our data reveal that phosphorylation and lysine acetylation follow distinct temporal programs during differentiation, which align with major biological transitions.

Phosphorylation–acetylation interplay has been described for transcriptional regulators such as MyoD and FOXO1, where phosphorylation-dependent signaling and acetylation cooperate to regulate stage-specific transcriptional activity during differentiation (28, 29). By capturing six differentiation stages and profiling more than 22,000 modified peptides, this study provides a comprehensive temporal, systems-level view of phosphorylation-acetylation dynamics during human myogenesis.

Globally, acetylation increased prominently at late differentiation stages, whereas phosphorylation underwent widespread bidirectional changes throughout the time course. These patterns suggest that late-stage transcriptional and metabolic reprogramming may be modulated predominantly through acetylation, while phosphorylation contributes more continuously to the dynamic regulation of signaling and cytoskeletal organization.

### Coordinated Regulation Among Kinases, Acetyltransferases, and Deacetylases

Coordinated changes in the predicted activities of kinases, acetyltransferases, and deacetylases underpin the global PTM trajectories observed during differentiation. KinSwing analysis identified temporally aligned activity patterns between kinases and acetylation-associated enzymes, several of which correspond to previously demonstrated phosphorylation events. Established regulatory relationships, including CSNK2A1–HDAC1/2, PRKAA1–HAT1, PRKD1–HDAC7, CDK1/2–KAT7, and SRC–SIRT2, were recapitulated by our data, supporting the validity of the activity-based inference approach.

In addition to these validated interactions, the analysis revealed candidate or context-dependent regulatory links. For example, SIRT2 displayed activity changes that were inconsistent with CDK1/2-mediated inhibitory phosphorylation, suggesting alternative regulatory inputs during myogenesis. Likewise, concordant temporal patterns between CDK4 and KAT2A, and the opposing activity profiles of PRKAA1 and SIRT2, highlight potential regulatory relationships that have not been mechanistically established in muscle differentiation.

The relationships identified here represent varying levels of supporting evidence for coordinated PTM regulation. While several kinase–acetylation enzyme pairs correspond to previously demonstrated phosphorylation-dependent interactions, others are supported primarily by concordant temporal activity patterns or functional associations. Likewise, spatially proximal phosphorylation and acetylation sites on dual-modified proteins identify candidate PTM integration regions but do not establish direct mechanistic crosstalk. These findings should therefore be interpreted as hypothesis-generating regulatory candidates for future biochemical and functional investigation.

Together, these findings suggest that phosphorylation and lysine acetylation are integrated through both established kinase–enzyme relationships and differentiation-specific regulatory configurations, linking upstream signaling dynamics to transcriptional and metabolic remodeling.

### Coordinated PTM Regulation and the Metabolic Switch

Several biological processes central to myogenesis, including metabolic adaptation, cell-cycle exit, and structural remodeling, appear to be influenced by coordinated PTM regulation. Myoblast differentiation is accompanied by a metabolic transition marked by enhanced glycolytic activity and mitochondrial remodeling (30). Key regulators of this shift include PGC-1α, which coordinates mitochondrial gene expression in skeletal muscle (31), and sirtuin deacetylases that regulate muscle metabolic programs (32). PRKAA1, the catalytic α1 subunit of AMPK, functions as an energy sensor linking cellular metabolic state with transcriptional and mitochondrial programs during muscle differentiation (33).

Our data are consistent with a model in which phosphorylation–acetylation crosstalk may contribute to this metabolic reconfiguration. The coordinated decline in CDK4 and KAT2A activity indicates a potential connection between cell-cycle signaling and chromatin acetylation, although direct phosphorylation of KAT2A by CDK4 has not been demonstrated. In addition, PRKAA1–HAT1 co-regulation and the predicted SIRT2–PRKAA1–SRC axis highlights candidate nodes at which energy-sensing kinases may intersect with acetylation-dependent chromatin or metabolic control. Taken together, these observations support a temporal model in which early phosphorylation-driven signaling changes precede and potentially facilitate a later acetylation-associated phase of metabolic and chromatin remodeling during myogenesis.

### Cell-Cycle Exit as a Candidate PTM-Coordinated Checkpoint

In parallel with metabolic reprogramming, cell-cycle withdrawal is required for myogenic commitment (15). Reduced predicted activity of CDK4–KAT2A and CDK1/2–KAT7 aligns with the established roles of CDKs in maintaining proliferative progression (15) and with the phosphorylation-dependent regulation of acetyltransferases such as KAT7 (34). These parallel shifts suggest that coordinated decreases in CDK and acetyltransferase activity may help link proliferation arrest with initiation of differentiation.

### Structural and Functional Implications for Lamin A/C and GAPDH

To examine how these coordinated regulatory programs act at the protein level, we evaluated dual-modified proteins as hypothesis-generating integration nodes.

Lamin A/C, encoded by *LMNA*, is essential for nuclear structure, chromatin anchoring, and mechanical stability (35). Lamin A/C is also involved in muscle differentiation and muscle disease (36). In this study, Lamin A/C exhibited dense clusters of regulated phosphorylation and acetylation sites. Several phosphorylation sites matched kinases with aligned activity predictions, and closely spaced phosphorylation–acetylation pairs in the head and tail domains showed inverse changes at late stages. Phosphorylation-dependent control of lamin assembly dynamics has been demonstrated before (37), and the extensive PTM clustering observed here further supports Lamin A/C as a candidate protein integrating PTM signaling during nuclear remodeling.

GAPDH displayed nine regulated modification sites positioned across catalytic, NAD^+^-binding, and oligomerization domains. Acetylation regulates metabolic enzyme function, including glycolytic enzymes such as GAPDH (11), and multiple PTMs contribute to GAPDH multifunctionality (38). Structural analyses define interface determinants that govern tetramer stability (39). Thus, the spatial clustering of regulated PTMs near interface regions identifies GAPDH as a potential PTM-sensitive metabolic node during differentiation.

### Therapeutic Implications and Future Applications

Multiple enzymes highlighted in this study, including sirtuins, HDACs, and AMPK, have been explored as therapeutic targets for muscle degeneration (40, 41, 42). Mapping stage-specific kinase-acetylation pathways expands the repertoire of potential intervention nodes. The temporal PTM workflow used here could also be applied to patient-derived cells or biopsy material to uncover PTM dysregulation in muscular dystrophies, sarcopenia, and cachexia.

### Methodological Considerations and Limitations

These interpretations must be considered in light of the methodological constraints inherent to PTM proteomics. PTM crosstalk predicted here is correlative. Low stoichiometry of dual-modified sites and their transient nature complicate detection (43). Furthermore, protein-abundance-corrected PTM measurements reflect relative modification levels normalized to protein abundance rather than direct measurements of absolute site occupancy. KinSwing predictions provide systems-level inference of candidate regulatory relationships; however, biochemical validation via mutagenesis, co-immunoprecipitation, structural assays, or PTM-mimetic studies will be required to confirm direct functional mechanisms. Limited annotations of acetyltransferase and deacetylase substrates also restrict definitive interpretation.

## Conclusion

By resolving phosphorylation and acetylation dynamics across six differentiation stages, this study establishes a temporal framework for understanding how layered post-translational regulation shapes myogenic progression. Distinct modification trajectories across signaling, metabolic, cytoskeletal, and nuclear pathways reveal how multiple layers of post-translational regulation coordinate the transition from myoblasts to early myotubes. Predicted enzyme activities and candidate PTM coordination relationships offer testable hypotheses for future mechanistic investigation.

## Data Availability

Raw mass spectrometry data have been deposited in the PRIDE repository under accession number PXD032760. Processed protein-level quantification tables, protein-corrected post-translational modification datasets, temporal clustering results, and KinSwing-predicted kinase, acetyltransferase, and deacetylase activity scores are provided in Supplementary Tables. This article contains supplementary data.

## Supplemental data

This article contains supplemental data (13, 17, 19, 20, 22, 23, 25, 26).

## Conflict of interest

The authors declare that they have no conflicts of interest with the contents of this article.

## Supplemental Data

Suplementary Figures S1–S7
