# Supplementary material for: A Temporal Phospho-acetylome Atlas of Human Myogenesis Identifies coordinated Post-Translational Regulation
Source: Mol Cell Proteomics. 2026 Jul 2;25(9):101619. doi: 10.1016/j.mcpro.2026.101619 (PMC13545378; doi:10.1016/j.mcpro.2026.101619)
Supplement: Supplementary file 1 [file mmc1.pdf]

Supplementary Figures

A temporal phospho-acetylome atlas of human myogenesis identifies coordinated post-translational regulation.

Lauren E. Smith[1], Christina E. Hagensen[1], Vasileios Tsiamis[1], Ashley J. van Waardenberg[2], Kamel Mamchaoui[3], Ole N. Jensen[1], Veit Schwämmle[1], Adelina Rogowska-Wrzesinska[1]*

[1] Department of Biochemistry and Molecular Biology, University of Southern Denmark, Odense, Denmark
[2] Australian Institute of Tropical Health and Medicine, James Cook University, Cairns, Australia
[3] Sorbonne University, INSERM, Institute of Myology, Center for Research in Myology, Paris, France

Present addresses:
Lauren E. Smith, Novo Nordisk, Kalundborg, Denmark; Christina E. Hagensen, Alphalyse A/S, Odense, Denmark; Vasileios Tsiamis, Department of Biotechnology and Biomedicine, Technical University of Denmark, Kgs. Lyngby, Denmark; Ashley J. van Waardenberg, i-Synapse PTY LTD, Cairns, Australia.

* **Correspondence:** adelinar@bmb.sdu.dk

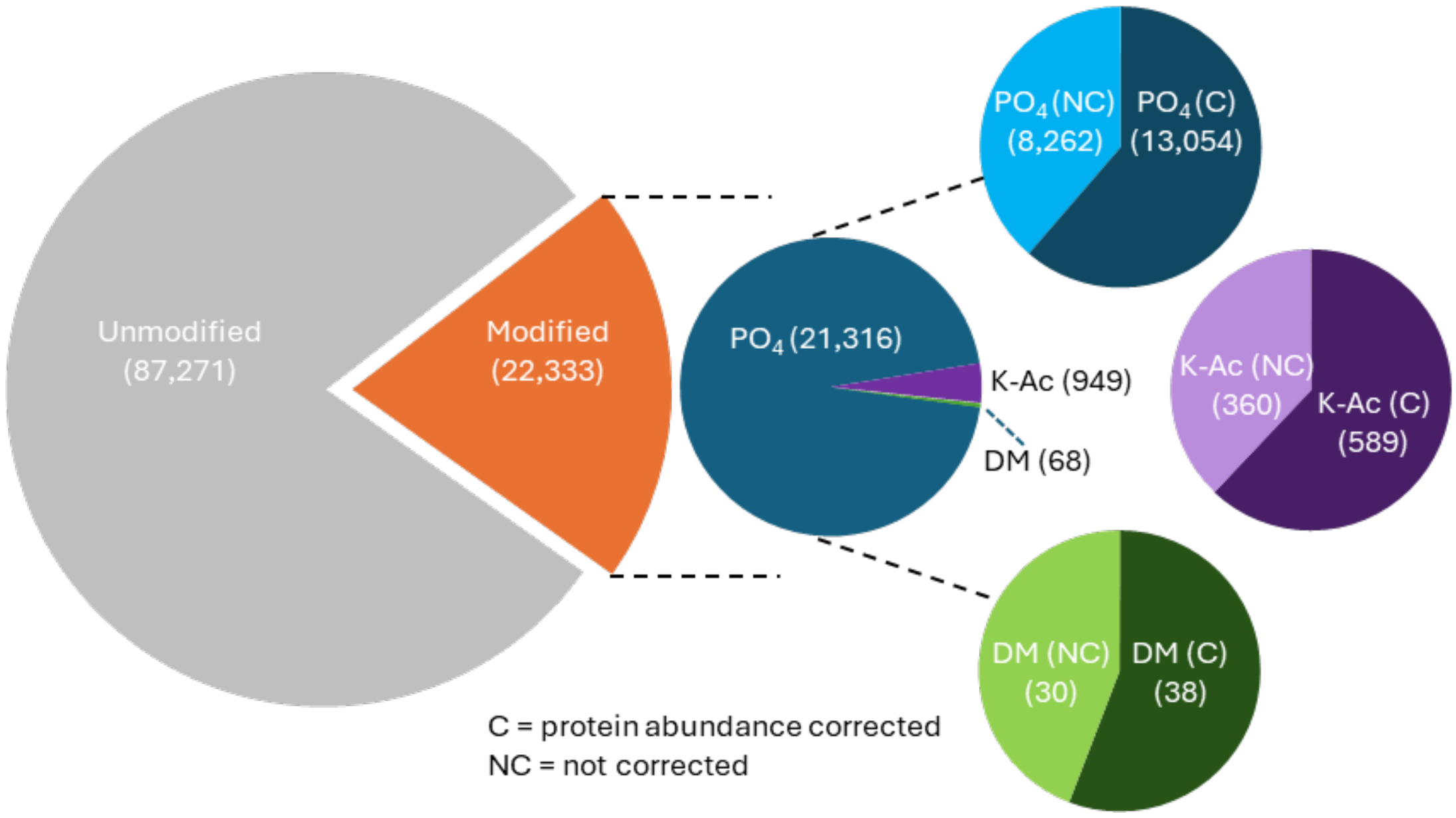

Figure S1. Overview of modified peptide identification and protein abundance correction. Distribution of unmodified and modified peptides identified across the dataset, and breakdown of phosphorylated (PO_4), lysine-acetylated (K-Ac), and dual-modified (DM) peptides. Modified peptides are shown as identified before correction (NC, not corrected) and retained following correction for underlying protein abundance using VIQoR (C, corrected). Numbers indicate peptide counts in each category.

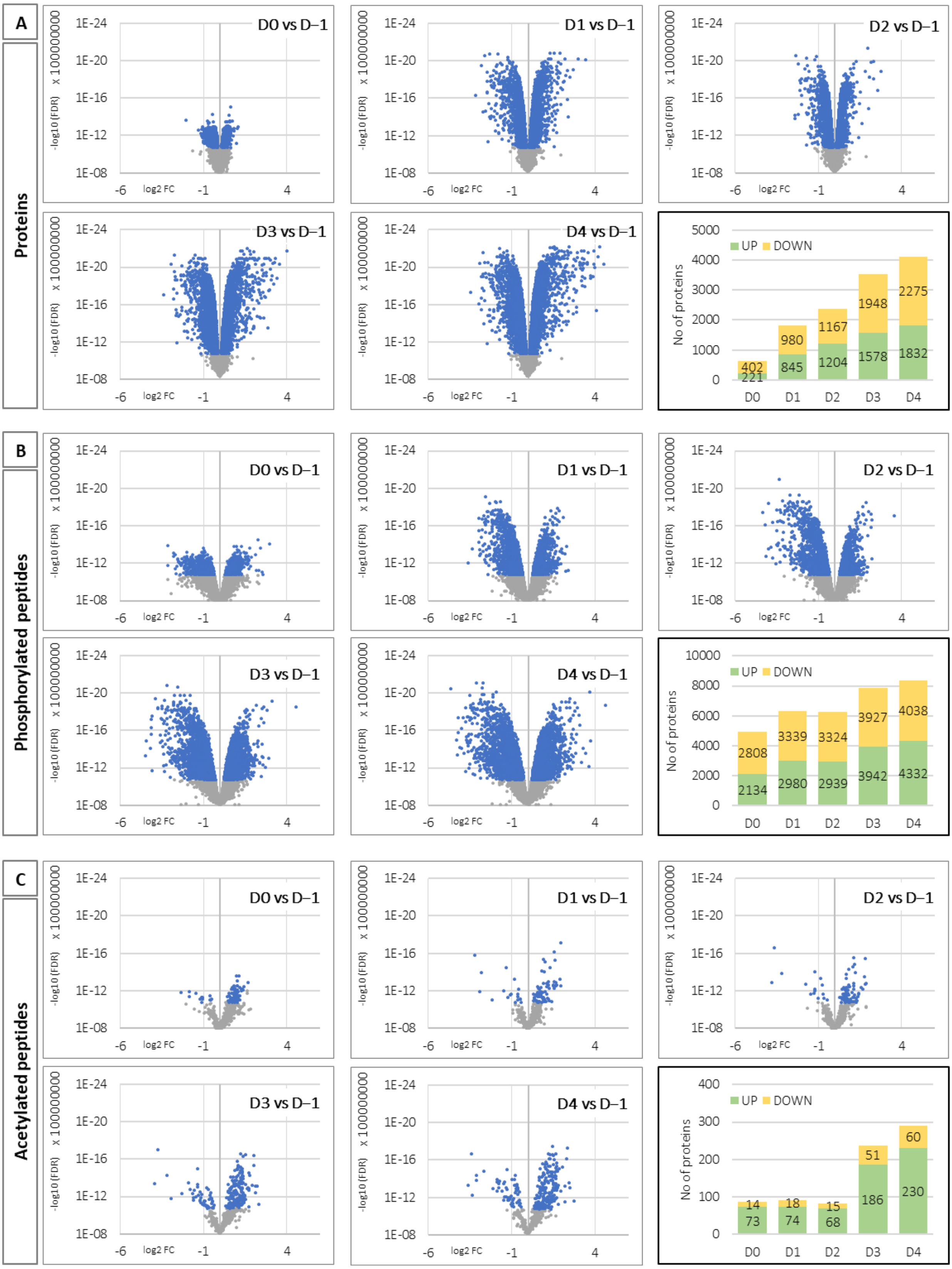

Figure S2. Volcano plot analysis of regulated proteins and modified peptides during myogenesis. Volcano plots show −log10(FDR) versus log2 fold changes for (A) proteins, (B) phosphorylated peptides, and (C) acetylated peptides at each differentiation stage relative to Day −1 (D−1). Significance was defined as FDR < 0.002 and |log2 fold change| ≥ 0.38. Blue points indicate significant changes; grey points indicate non-significant changes. Adjacent bar charts summarize the numbers of significantly increased and decreased proteins or modified peptides at each time point. Proteins and phosphorylated peptides showed bidirectional regulation throughout differentiation, whereas lysine-acetylated peptides predominantly increased at late stages (D3-D4).

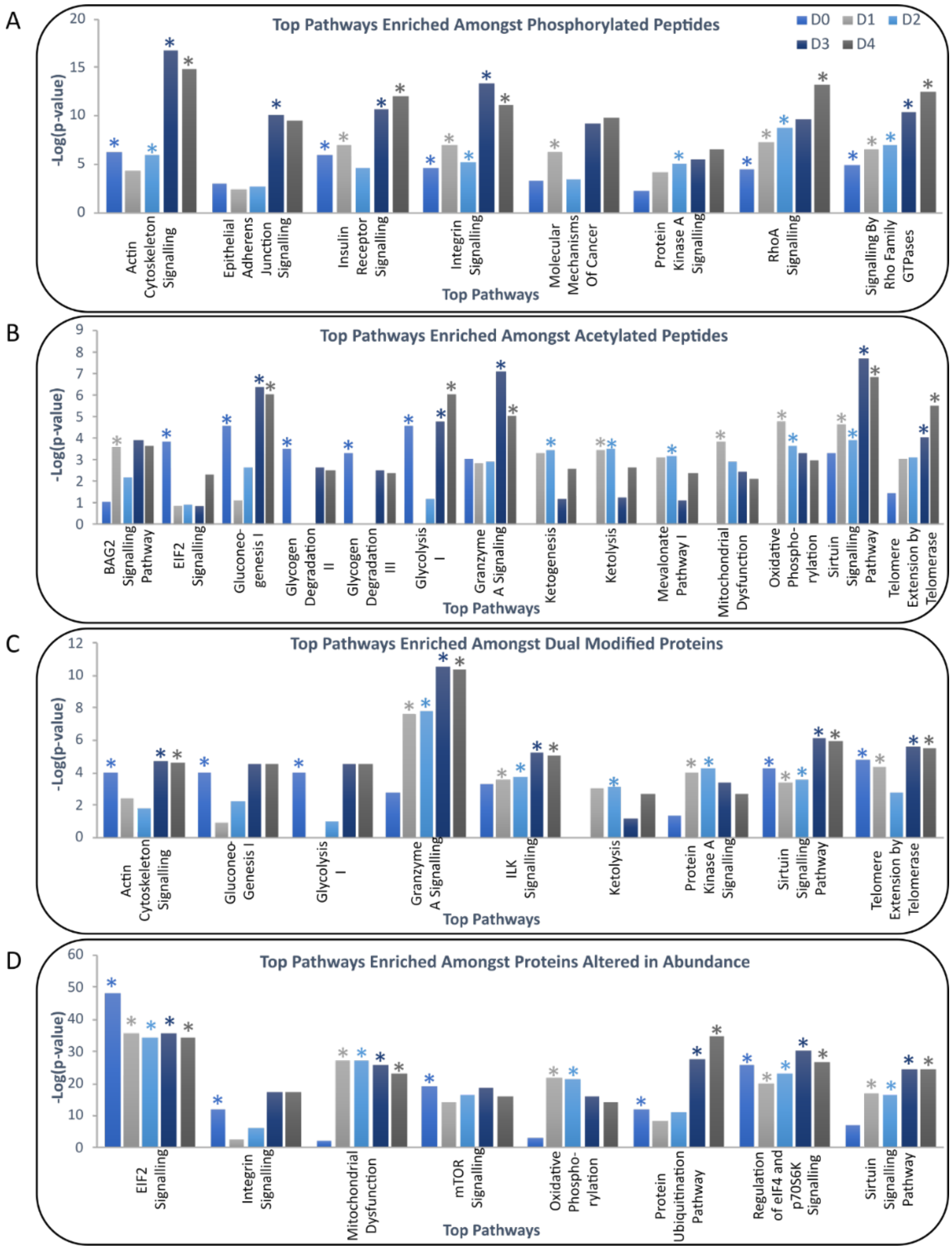

Figure S3. Top signaling pathways enriched at each time point during myogenesis. The top 5 pathways enriched at each time point compared to D-1 were identified using Ingenuity Pathway Analysis for: (A) Phosphorylated peptides. Phosphorylation is highly involved in cytoskeletal remodeling pathways throughout differentiation. (B) Acetylated peptides. Acetylation regulates metabolic pathways, particularly glycolysis/gluconeogenesis and oxidative phosphorylation, during the early-mid stages. (C) Proteins modified by both PTMs. Overlap between top pathways indicates coordinated regulation by phosphorylation and acetylation. (D) Total proteins with altered abundance. Signaling pathways controlling translation (EIF2, mTOR) are strongly enriched across differentiation. Key insights: Together, the data suggest phosphorylation primarily regulates cytoskeletal dynamics, while acetylation impacts metabolic pathways. Cross-talk may occur where these processes intersect, such as in glycolysis. Altered protein abundance also implicates translational control pathways in driving the differentiation program.

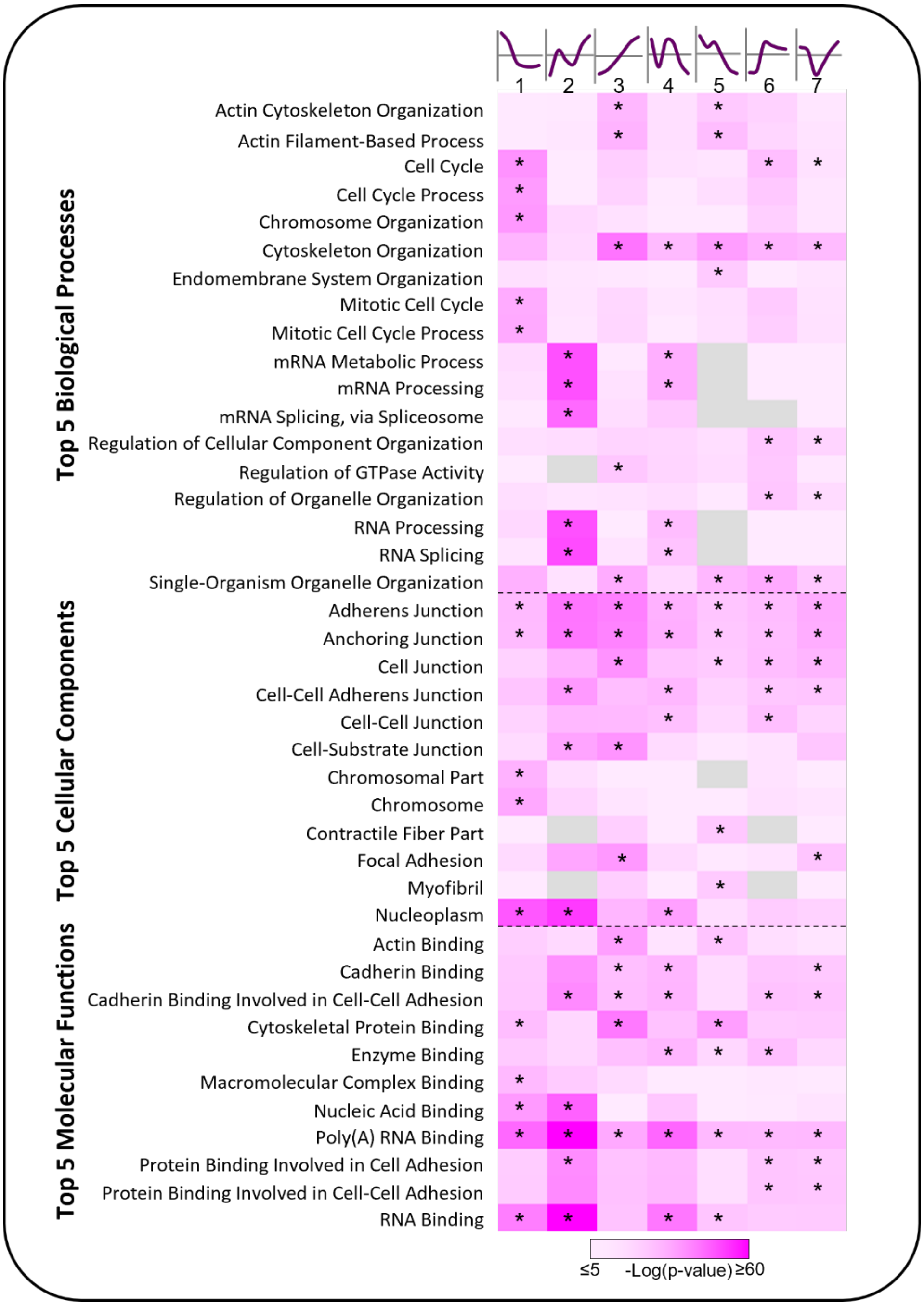

Figure S4. Gene Ontology term enrichment of phosphorylated peptide clusters. Phosphorylated peptides were clustered based on abundance changes over time (Figure S7A). Each cluster was analyzed for enriched GO terms using DAVID. The heatmap summarizes the top 5 enriched terms (FDR ≤0.05) within each of the 3 GO categories - Biological Process, Cellular Component, and Molecular Function - for each cluster (*). Colors indicate enrichment significance based on the -log EASE score, with darker shades indicating greater significance. Grey means the term was not enriched in that cluster. Key insights: Cluster 1 is enriched for cell-cycle-related processes, suggesting decreased proliferation. Actin/cytoskeleton-associated terms are enriched across clusters, implicating phosphorylation in remodeling. mRNA/RNA-related terms enriched in multiple clusters highlight transcriptional/translational control. This analysis links phosphorylation dynamics to specific biological functions during myogenic differentiation.

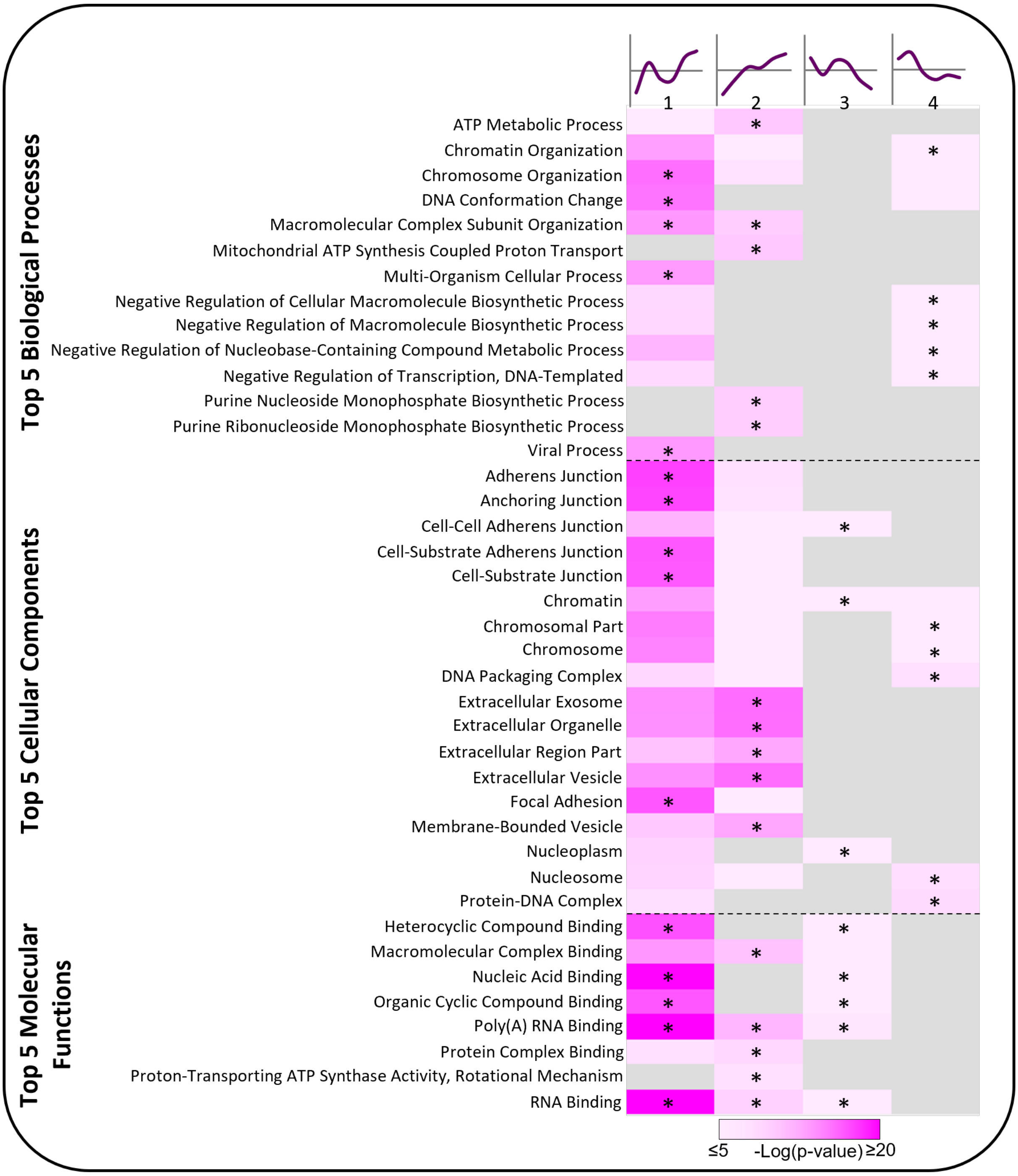

Figure S5. Gene Ontology term enrichment of acetylated peptide clusters. Acetylated peptides were clustered based on abundance changes (Figure S7B). GO term enrichment was analyzed using DAVID. The heatmap summarizes the top 5 enriched terms (FDR ≤0.05) within each GO category for each cluster (*). Colors indicate enrichment significance based on -log EASE score. Grey means the term was not enriched. Key insights: Cluster 1 shows enrichment for DNA- and chromosome-related processes, reflecting acetylation dynamics. Cluster 2 is enriched for ATP metabolism terms, linking acetylation to metabolic regulation. Cluster 4 involves negative transcription regulation, localized to DNA/chromosomes. Clusters 3 and 4 lacked significantly enriched terms in some categories (as noted in the legend). This analysis links distinct acetylation dynamics to gene expression and metabolic processes over myogenesis. The lack of enrichment in some clusters/categories suggests more complex regulations.

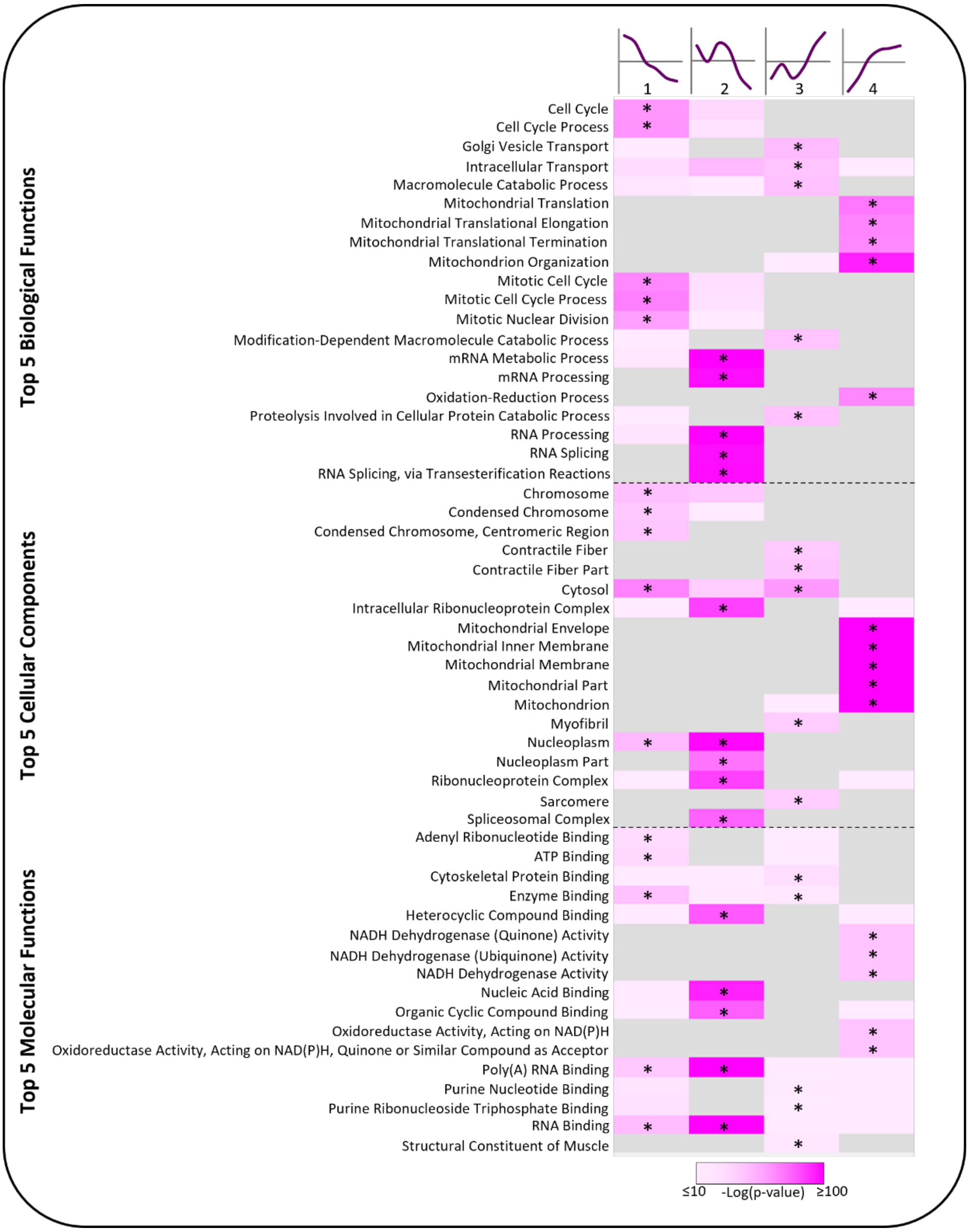

Figure S6. Gene Ontology term enrichment of protein abundance clusters. Proteins were clustered based on abundance changes over time (Figure S7C). GO term enrichment was analyzed using DAVID. The heatmap summarizes the top 5 enriched terms (FDR ≤0.05) within each GO category for each cluster (*). Colors indicate enrichment significance based on -log EASE score. Grey indicates the term was not enriched. Key insights: Cluster 1 involves cell-cycle processes, suggesting decreased proliferation. Cluster 4 implicates mitochondrial components and NADH activity, linking increased abundance to metabolic regulation. Clusters 2 and 3 show bidirectional changes linked to distinct functions. This analysis links dynamic protein expression patterns to biological processes driving myogenic differentiation, such as cell cycle exit and metabolic remodeling. Distinct clusters suggest stage-specific protein functions.

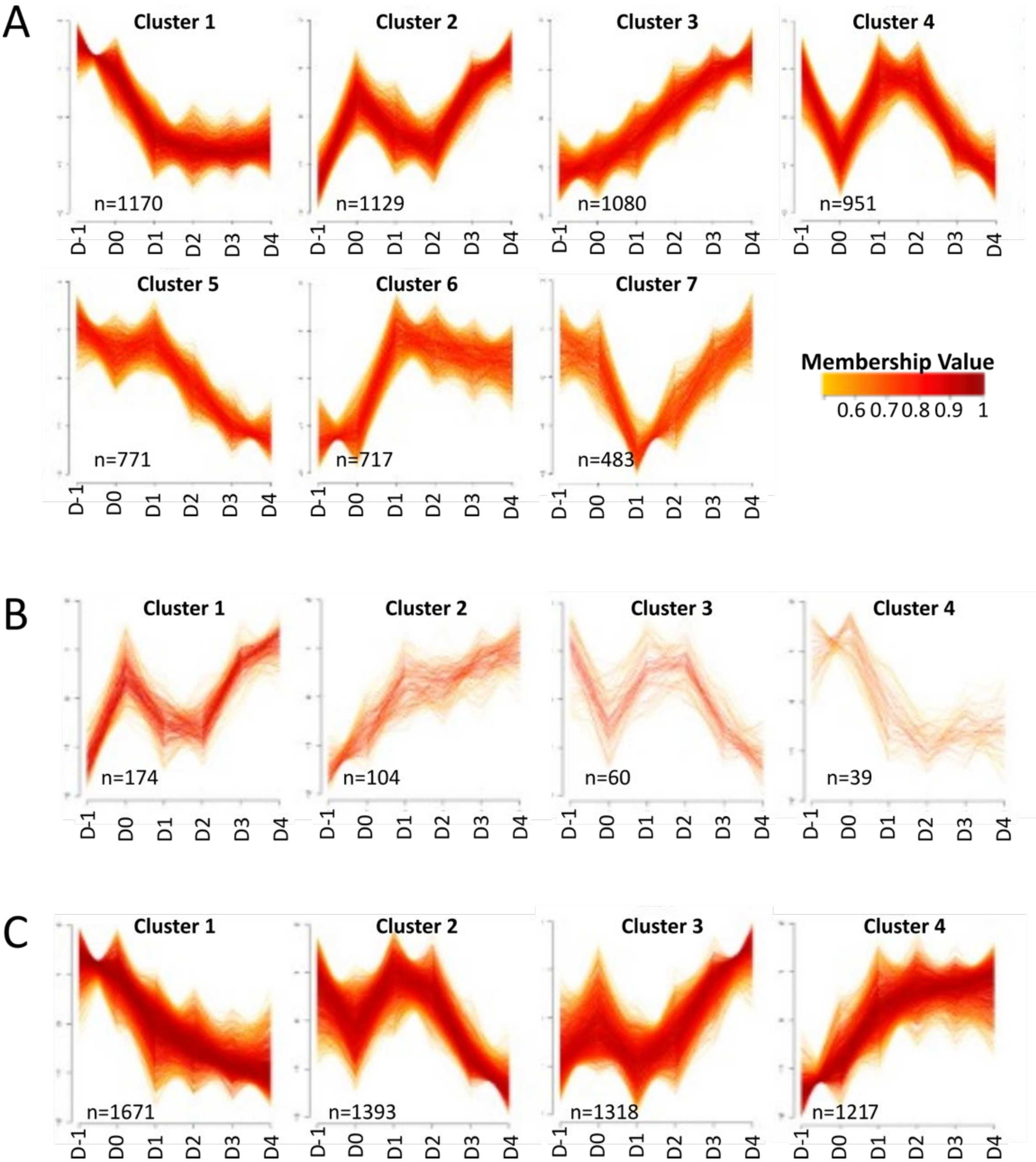

Figure S7. Temporal clustering of modified peptides and proteins across the differentiation time course. Modified peptides and proteins were clustered across time points using VSClust fuzzy c-means clustering. (A) Phosphorylated peptides are clustered into seven distinct patterns. (B) Acetylated peptides clustered into four patterns. (C) Proteins are clustered into four patterns. The x-axis represents differentiation time points (D-1 to D4), and the y-axis shows normalized abundance. Each line represents a peptide with a distinct temporal expression profile. Numbers (n) indicate the number of peptides assigned to each cluster.
