# Supplementary material for: A Temporal Phospho-acetylome Atlas of Human Myogenesis Identifies coordinated Post-Translational Regulation
Source: Mol Cell Proteomics. 2026 Jul 2;25(9):101619. doi: 10.1016/j.mcpro.2026.101619 (PMC13545378; doi:10.1016/j.mcpro.2026.101619)
Supplement: Supplementary file 2 [file mmc3.pdf]

Supplementary Methods

A temporal phospho-acetylome atlas of human myogenesis identifies coordinated post-translational regulation.

Lauren E. Smith[1], Christina E. Hagensen[1], Vasileios Tsiamis[1], Ashley J. van Waardenberg[2], Kamel Mamchaoui[3], Ole N. Jensen[1], Veit Schwämmle[1], Adelina Rogowska-Wrzesinska[1]*

[1] Department of Biochemistry and Molecular Biology, University of Southern Denmark, Odense, Denmark
[2] Australian Institute of Tropical Health and Medicine, James Cook University, Cairns, Australia
[3] Sorbonne University, INSERM, Institute of Myology, Center for Research in Myology, Paris, France

Present addresses:
Lauren E. Smith, Novo Nordisk, Kalundborg, Denmark; Christina E. Hagensen, Alphalyse A/S, Odense, Denmark; Vasileios Tsiamis, Department of Biotechnology and Biomedicine, Technical University of Denmark, Kgs. Lyngby, Denmark; Ashley J. van Waardenberg, i-Synapse PTY LTD, Cairns, Australia.

* **Correspondence:** adelinar@bmb.sdu.dk

Reagents and Media Components

Unless stated otherwise, all reagents were obtained from Sigma-Aldrich (St. Louis, MO, USA). Media components for the cultivation of KM155C25 human satellite cells included DMEM with GlutaMAX containing 4.5 g/L glucose (Gibco 61965-026), Medium 199 with GlutaMAX containing 1.0 g/L glucose (Gibco 41150-020), fetal bovine serum (Sigma F7524, Lot BCBV7611), insulin (Sigma 91077C), penicillin/streptomycin (Gibco 15140122), fetuin (Sigma F2379), human EGF (Gibco PHG0311), human bFGF (Gibco PHG0026), and dexamethasone (Sigma D4902). All solvents were LC-MS grade unless specified.

Cell Culture and Differentiation

KM155C25 cells were grown in a humidified incubator at 37°C and 5% CO_2. Growth medium consisted of four parts DMEM and one part Medium 199 supplemented with 20% fetal bovine serum, 25 µg/mL fetuin, 5 ng/mL hEGF, 0.5 ng/mL bFGF, 5 µg/mL insulin, 0.5% penicillin/streptomycin, and 0.2 µg/mL dexamethasone. Cells were maintained in T75 flasks and passaged after reaching approximately 70% confluence using standard trypsinization procedures.

For differentiation, cells were seeded to reach 90-100% confluence and subsequently switched to differentiation medium composed of DMEM supplemented with 10 µg/mL insulin and penicillin/streptomycin. Samples were harvested at six defined stages of the myogenic program: D-1 (proliferating cells at approximately 70% confluence), D0 (fully confluent undifferentiated myoblasts), and D1 through D4 at 24-hour intervals from the onset of differentiation. Each time point was collected in three independent biological replicates. For immunostaining, cells were plated on glass slides coated with 0.5% gelatin and processed accordingly.

Protein Extraction and FASP Digestion

Cells were washed twice with cold PBS and lysed in buffer containing 50 mM TEAB (pH 8.0), 1% sodium deoxycholate, 10 mM TCEP, 40 mM chloroacetamide, and protease and phosphatase inhibitors (Roche). Lysates were heated to 80°C for 10 minutes, vortexed for 5 minutes, and sonicated 3 times for 15 seconds at 40% output, with cooling intervals on ice. After centrifugation at 14,000 × g for 10 minutes, the clarified supernatant was transferred to new tubes, and protein concentration was determined by tryptophan fluorescence, as described by (1).

Protein digestion was performed using the filter-aided sample preparation (FASP) method on 30 kDa molecular weight cut-off filters (Vivacon 500, Sartorius). A total of 350 µg of protein was loaded per filter, and the filters were centrifuged at 14,000 × g for 15 minutes. The protein was washed three times with 50 mM ammonium bicarbonate and subsequently digested overnight at 37 °C with methylated trypsin (2) at an enzyme-to-protein ratio of 1:25. A second digestion step was performed the following day using an enzyme-to-protein ratio of 1:50 for 3 hours at 37°C. Peptides were collected by centrifugation, and filters were washed with additional ammonium bicarbonate. The combined peptide eluates were lyophilized.

Peptide Desalting and TMT Labeling

Lyophilized peptides were resuspended in 0.1% trifluoroacetic acid and desalted on Oasis HLB solid-phase extraction cartridges (Waters). Peptides were eluted using 80% acetonitrile with 0.1% TFA, dried, and dissolved in 0.1 M TEAB for TMT labeling.

Peptides (100 µg per sample) were labeled with TMT10plex reagents (Thermo Fisher Scientific) according to the manufacturer's instructions. Labeling reactions were incubated for one hour at room temperature and quenched with 5% hydroxylamine. Samples were combined into two TMT 10-plex experiments, with both plexes including a 131 pooled reference channel created from equal aliquots of all samples. The multiplex structure and channel assignments are summarized in Supplementary Table S10. The pooled TMT-labeled samples were dried by vacuum centrifugation before enrichment.

Phosphopeptide Enrichment Using Titanium Dioxide

Phosphorylated peptides were enriched using sequential incubations with titanium dioxide beads. Peptides were dissolved in a loading buffer composed of 80% acetonitrile, 5% TFA, and 1 M glycolic acid. The peptide solution was incubated first with 6 mg of TiO_2 beads for 5 minutes at room temperature with gentle shaking. After centrifugation for 30 seconds, the supernatant was transferred to a fresh tube and incubated with 3 mg of TiO_2 beads, then with 1.5 mg, under the same conditions.

Following enrichment, the beads were washed with loading buffer, then with 80% acetonitrile containing 1% TFA, and finally with 20% acetonitrile containing 0.5% TFA. Phosphorylated peptides were eluted twice using 1% ammonium hydroxide at pH 11 for 10 minutes with shaking, dried by vacuum centrifugation, and desalted using C18/POROS R3 reversed-phase micro-columns.

Acetyl-Lysine Enrichment Using Immunoaffinity Purification

The supernatants from TiO_2 enrichment were used for the subsequent immunoaffinity purification of acetylated peptides using the PTMScan® Acetyl-Lysine Motif Kit (Cell Signaling Technology, catalogue number 13416). Peptides were dissolved in 1× IAP buffer, clarified by centrifugation at 10,000 × g for 5 minutes at 4°C, and incubated with antibody-conjugated beads for 2 hours at 4°C with rotation. The supernatant and the first wash were collected for analysis of non-modified peptides.

The beads were washed three times with IAP buffer and a further three times with ice-cold water. Bound acetylated peptides were eluted twice using 0.15% TFA for 10 minutes at room temperature, centrifuged to separate beads, and desalted using C18/POROS R3 micro-columns before LC-MS/MS analysis.

High-pH Reverse-Phase Fractionation

Phosphorylated and non-modified peptide samples were fractionated using high-pH reversed-phase chromatography on a Dionex UltiMate 3000 nanoLC system. Samples were dissolved in buffer containing 20 mM ammonium formate at pH 9.3 and separated over a 97-minute gradient from 2% to 50% acetonitrile in the same buffer. Fractions were collected every 151 seconds, combined into 20 fractions, and dried by vacuum centrifugation.

LC-MS/MS Analysis

Peptides were analyzed on a Q Exactive HF-X mass spectrometer (Thermo Fisher Scientific) coupled to a nanoflow LC system equipped with a home-packed C18 analytical column (50 cm × 100 µm internal diameter, 1.9 µm particle size, 120 Å pore size). The column was maintained at 50°C and operated at a flow rate of 475 nL/min. For acetylated and non-modified peptides, a 55-minute gradient ranging from 8% to 45% buffer B (99.99% acetonitrile, 0.01% formic acid) was used. For phosphorylated peptides, a 5% to 45% gradient over 55 minutes was applied.

MS1 spectra were acquired at 120,000 resolution with an AGC target of 3×10^6 ions, a maximum injection time of 50 ms, and a scan range of 350-1500 m/z. MS2 acquisition followed a Top20 data-dependent method with a resolution of 45,000, an AGC target of 1×10^5, a maximum injection time of 100 ms, and higher-energy collisional dissociation using a collision energy of 32 for acetylated and non-modified peptides and 34 for phosphorylated peptides. A dynamic exclusion window of 20 seconds was applied.

Data Processing Using Proteome Discoverer

Raw data were processed in Proteome Discoverer version 2.3 using Mascot as the search engine. The SwissProt human database (taxonomy 9606; release March 2020; 20,431 protein entries) was used for identification. Search parameters included a precursor mass tolerance of 10 ppm, a fragment mass tolerance of 0.05 Da, and trypsin digestion with up to 4 missed cleavages. Carbamidomethylation of cysteine was set as a static modification. In contrast, phosphorylation of serine, threonine, and tyrosine, acetylation of lysine, and TMT10plex labeling of lysine residues and peptide N-termini were included as variable modifications.

PSMs were filtered using Percolator to achieve a 1% false discovery rate. Site localization was assessed using ptmRS, and only sites with localization probabilities of at least 90% were retained. Reporter ions were integrated using a 30 ppm window. Reporter ion quantification was performed using the Proteome Discoverer Reporter Ions Quantifier node with HCD MS/MS-based TMT quantification. Reporter abundance calculation used the Proteome Discoverer automatic reporter abundance mode. An average reporter signal-to-noise threshold of 6 was applied, and the co-isolation threshold was set to 100. Isotopic impurity correction was not applied during reporter ion quantification. Protein-level quantification was based on unique peptides only using protein-group-based peptide assignment. PSMs missing the 131 reference channel or containing shared reporter labels were excluded. Data were normalized by subtracting $\log_2$-transformed intensities of the reference channel, after which the two TMT plexes were combined for downstream analysis.

Protein Inference and PTM Correction with VIQoR

Protein inference and PTM abundance correction were performed using VIQoR, which applies probabilistic peptide-to-protein assignment and weighted averaging to estimate protein abundances. Input data were log-transformed before VIQoR processing(3). The analysis included 18 quantitative columns corresponding to six biological conditions, and phosphorylation and lysine acetylation were selected as the quantified modification types. Median normalization was applied during VIQoR processing, and protein inference was performed using the strict inference setting. Modified peptide

intensities were corrected for changes in the corresponding protein abundance to allow interpretation of modification-specific regulation.

Statistical Analysis Using Polystest

Differential regulations were assessed using the Polystest framework, which integrates multiple statistical tests and includes the Miss test to account for non-random missing values (4). A false discovery rate of 0.002 was applied to both peptides and proteins, yielding an overall FDR of approximately 0.01. Fold-change thresholds of ±1.3 were selected based on the observed replicate variability, which had a mean coefficient of variation below 12% across sample groups. All normalized and Polystest-processed datasets are provided in Supplementary Tables S1 and S2.

Peptide-level statistical significance was assessed using the Polystest Miss test, applied independently to phosphorylated, lysine-acetylated, and dual-modified peptides. Significantly regulated peptides were defined by an absolute $\log_2$ fold change ≥ 0.38 (corresponding to a fold change ≥ 1.3) and an adjusted p-value (FDR) < 0.002. This thresholding resulted in an approximate dataset-level false discovery rate of ~0.01 after multiple testing correction. Peptide intensities corrected for underlying protein abundance using VIQoR are denoted as corrected values, whereas uncorrected peptide intensities are reported as not corrected.

Clustering and Gene Ontology Enrichment

Temporal expression and modification patterns were analyzed using variance-sensitive fuzzy c-means clustering implemented in VSClust (5). The number of clusters was chosen based on internal VSClust optimization criteria. Gene Ontology enrichment was performed using DAVID (6), analyzing Biological Process, Molecular Function, and Cellular Component terms, with significance defined as Benjamini-Hochberg-adjusted p ≤ 0.05. Corresponding clustering and enrichment results are presented in Supplementary Figures S4-S7.

Pathway enrichment analysis

Pathway enrichment analysis was performed using Ingenuity Pathway Analysis (IPA, Qiagen). Separate analyses were conducted for phosphorylated peptides, lysine-acetylated peptides, dual-modified peptides, and proteins exhibiting significant abundance changes. For peptide-based analyses, only peptides passing localization probability filtering (≥90%) and protein-abundance correction using VIQoR were included. For each dataset, significantly regulated entities ($|\log_2$ fold change$| \geq 0.38$ and FDR < 0.002) were analyzed independently at each differentiation time point relative to Day −1.

IPA enrichment was assessed using right-tailed Fisher’s exact tests, with Benjamini–Hochberg correction applied to control for multiple testing. Pathways with adjusted p-values ≤ 0.01 were considered significantly enriched. The IPA knowledge base corresponding to the analysis date was used as background. No directionality filtering was applied, and pathway enrichment was based solely on membership overlap. Complete pathway results are provided in Supplementary Tables S3–S6.

KinSwing Enzyme Activity Predictions

KinSwingR was applied to protein-abundance-corrected phosphorylation and lysine-acetylation datasets. Input substrates for modeling kinase position weight matrices were obtained from PhosphoSitePlus using human substrate annotations (downloaded 20/11/2019) (7). Substrate relationships for acetyltransferases and deacetylases were obtained from the curated enzyme–substrate dataset reported by Yu et al. (8). In all cases, substrate sequences were centered to a total length of 15 amino acids prior to analysis. KinSwing scores were computed for each differentiation time point relative to Day −1. Statistical significance was assessed using permutation-based p-values derived from 1,000 random perturbations of the motif–substrate network, and enzymes with $p \leq 0.05$ were retained. Hierarchical clustering of significant enzymes was performed to group enzymes with similar temporal activity profiles. Full KinSwing scores and clustering assignments are provided in Supplementary Table S8.

Supplementary Table Legends

Table S1. Reproducibility of quantitative measurements across time points. The table reports mean and median coefficients of variation (CV, %) across biological replicates for phosphorylated peptides, lysine-acetylated peptides, and proteins at each differentiation time point. Based on the observed technical variability, a fold change threshold of 1.3 (approximately 30%) was considered biologically significant for downstream analyses.

Table S2 (A-B). Quantitative analysis of post-translationally modified peptides during myoblast differentiation. This table lists quantified post-translationally modified peptides identified across the myoblast differentiation time course, including phosphorylated, lysine-acetylated, and dual-modified peptides. Separate sub-tables are provided for protein-abundance-corrected modified peptide values (Table S2A) and corresponding non-corrected modified peptide abundance values prior to VIQoR correction (Table S2B). Table S2A includes protein group assignment, protein and gene annotation, normalized modification levels for each biological replicate at each time point (D−1 to D4), $\log_2$ fold changes relative to D−1, and corresponding false discovery rates (FDR) derived from unified peptide-level statistical testing. Table S2B contains the corresponding raw modified peptide abundance values, enabling direct comparison between corrected PTM regulation and absolute changes in modified peptide abundance. Rows between Table S2A and Table S2B correspond directly and can be matched using the modified peptide sequence and counterpart sequence columns. Details of peptide filtering, protein abundance correction, and statistical analysis are described in the Supplementary Methods.

Table S3. Quantitative protein abundance changes during myoblast differentiation. This table reports quantitative measurements of protein abundance across the myoblast differentiation time course. For each protein group, protein and gene annotation, peptide coverage, normalized protein abundance values for each biological replicate at each time point (D–1 to D4), $\log_2$ fold changes relative to Day −1, and corresponding false discovery rates (FDR) derived from unified protein-level statistical testing are provided. Details of protein quantification, normalization, and statistical analysis are described in the Supplementary Methods.

Table S4 (A-D). Pathway enrichment analysis across post-translational modification classes and protein abundance changes. This table summarizes Ingenuity Pathway Analysis (IPA) results for enriched canonical pathways identified across the myoblast differentiation time course. Separate sub-tables are provided for phosphorylated peptides (Table S4A), lysine-acetylated peptides (Table S4B), dual-modified peptides (Table S4C), and proteins exhibiting significant abundance changes (Table S4D). For each pathway, the differentiation time point, pathway name, enrichment significance, pathway ratio, and matched molecules are reported. Interpretation of the pathway

Table S5 (A-C). Gene Ontology enrichment analysis of phosphorylation clusters. This table reports Gene Ontology (GO) term enrichment results for clusters of phosphorylated peptides identified by temporal clustering. Enriched GO terms are listed separately for the Biological Process (Table S5A), Cellular Component (Table S5B), and Molecular Function (Table S5C) categories. For each GO term, identifiers, term names, enrichment significance, enrichment score, and associated gene counts are provided. Corresponding visual summaries are shown in Supplementary Figure S4.

Table S6 (A-C). Gene Ontology enrichment analysis of acetylation clusters. This table reports Gene Ontology (GO) term enrichment results for clusters of lysine-acetylated peptides identified by temporal clustering. Enriched GO terms are listed separately for the Biological Process (Table S6A), Cellular Component (Table S6B), and Molecular Function (Table S6C) categories. For each GO term, identifiers, term names, enrichment significance, enrichment score, and associated gene counts are provided. Corresponding visual summaries are shown in Supplementary Figure S5.

Table S7 (A-C). Gene Ontology enrichment analysis of protein abundance clusters. This table reports Gene Ontology (GO) term enrichment results for protein clusters exhibiting similar temporal abundance profiles during myoblast differentiation. Enriched GO terms are listed separately for the Biological Process (Table S7A), Cellular Component (Table S7B), and Molecular Function (Table S7C) categories. For each GO term, identifiers, term names, enrichment significance, enrichment score, and associated gene counts are provided. Corresponding visual summaries are shown in Supplementary Figure S6.

Table S8 (A-C). KinSwing-based inference of enzyme activity changes during myoblast differentiation. This table reports inferred changes in enzyme activity involved in phosphorylation- and acetylation-related signaling across the myoblast differentiation time course, as estimated using KinSwing analysis. Enzymes are grouped by functional class, including kinases (Table S8A), lysine acetyltransferases (Table S8B), and deacetylation-associated enzymes (Table S8C). For each enzyme, swing scores and corresponding one-sided p-values are reported for each differentiation time point relative to Day −1, indicating the direction and magnitude of predicted activity changes. Details of the KinSwing analysis are described in the Supplementary Methods.

Table S9. Proteins carrying both phosphorylation and acetylation sites during myoblast differentiation. This table lists site-resolved post-translationally modified peptides derived from proteins that harbor both phosphorylation and lysine-acetylation sites across the myoblast differentiation time course. For each modified peptide, modification type and site, protein assignment used for site localization, protein and gene annotation, $\log_2$ fold changes relative to Day −1 at each time point, and corresponding false discovery rates (FDR) from unified peptide-level statistical testing are reported. Details of peptide filtering and statistical analysis are described in the Supplementary Methods.

Table S10. TMT10plex experimental design and channel assignment. The table summarizes the allocation of differentiation stages across the two TMT10plex experiments used in this study. Each multiplex included a pooled mixed reference channel (131) composed of equal peptide aliquots from all samples, which was used for cross-plex normalization. Samples from different differentiation stages were distributed across both plexes to minimize stage-specific batch effects.

References

1. Wisniewski, J. R., and Gaugaz, F. Z. (2015) Fast and sensitive total protein and Peptide assays for proteomic analysis. *Anal Chem* 87, 4110-4116
2. Heissel, S., Frederiksen, S. J., Bunkenborg, J., and Højrup, P. (2019) Enhanced trypsin on a budget: Stabilization, purification and high-temperature application of inexpensive commercial trypsin for proteomics applications. *PLoS One* 14, e0218374
3. Tsiamis, V., and Schwämmle, V. (2022) VIQoR: a web service for visually supervised protein inference and protein quantification. *Bioinformatics* 38, 2757-2764
4. Schwämmle, V., Hagensen, C. E., Rogowska-Wrzesinska, A., and Jensen, O. N. (2020) PolySTest: Robust Statistical Testing of Proteomics Data with Missing Values Improves Detection of Biologically Relevant Features. *Mol Cell Proteomics* 19, 1396-1408
5. Schwämmle, V., and Jensen, O. N. (2018) VSClust: feature-based variance-sensitive clustering of omics data. *Bioinformatics* 34, 2965-2972
6. Sherman, B. T., Hao, M., Qiu, J., Jiao, X., Baseler, M. W., Lane, H. C., Imamichi, T., and Chang, W. (2022) DAVID: a web server for functional enrichment analysis and functional annotation of gene lists (2021 update). *Nucleic Acids Res* 50, W216-w221
7. Hornbeck, P. V., Kornhauser, J. M., Tkachev, S., Zhang, B., Skrzypek, E., Murray, B., Latham, V., and Sullivan, M. (2012) PhosphoSitePlus: a comprehensive resource for investigating the structure and function of experimentally determined post-translational modifications in man and mouse. *Nucleic Acids Res* 40, D261-270
8. Yu, K., Zhang, Q., Liu, Z., Du, Y., Gao, X., Zhao, Q., Cheng, H., Li, X., and Liu, Z.-X. (2019) Deep learning based prediction of reversible HAT/HDAC-specific lysine acetylation. *Briefings in Bioinformatics* 21, 1798-1805
